# *Euonymus alatus* Twig Extract Protects against Scopolamine-Induced Changes in Brain and Brain-Derived Cells via Cholinergic and BDNF Pathways

**DOI:** 10.3390/nu15010128

**Published:** 2022-12-27

**Authors:** Pallavi Gurung, Rajeev Shrestha, Junmo Lim, Til Bahadur Thapa Magar, Han-Hyuk Kim, Yong-Wan Kim

**Affiliations:** 1 Dongsung Cancer Center, Dongsung Biopharmaceutical, Daegu 41061, Republic of Korea; 2 Medical Convergence Textile Center, Gyeongbuk Technopark, Gyeongsan 38412, Republic of Korea

**Keywords:** cognitive deficits, scopolamine, brain-derived neurotrophic factor (BDNF), catechin, acetylcholine esterase, hippocampus

## Abstract

In the current study, the therapeutic and preventive effects of *Euonymus alatus* (EA) twig extract were investigated in a mouse model of cognitive deficit and B35 cells. Twig extract 1 was extracted with 70% ethanol and later twig extract 2 was extracted through liquid-liquid extraction with 70% ethanol and hexane. EA twig 2 (300 mg/kg) along with the standard drug donepezil (5 mg/kg) were orally administered to the mice for 34 days. Scopolamine was given intraperitoneally for 7 days. Administration of EA twig extract 2 significantly improved the passive avoidance test (PAT) in mice. EA twigs extract also restored the scopolamine-reduced brain-derived neurotrophic factor (BDNF)/extracellular regulated kinase (ERK)/cyclic AMP responsive element binding protein (CREB) signaling in B35 cells and the mouse hippocampus. In addition, EA twig extract significantly inhibited the acetylcholine esterase (AChE) activity in B35 cells in a dose-dependent manner. Chromatography and ESI MS analysis of EA twig extract revealed the presence of flavonoids; epicatechin, taxifolin, aromadendrin, and naringenin with catechin being the most abundant. These flavonoids exerted protective effects alone and had the possibility of synergistic effects in combination. Our work unmasks the ameliorating effect of EA twig extract 2 on scopolamine-associated cognitive impairments through the restoration of cholinergic systems and the BDNF/ERK/CREB pathway.

## 1. Introduction

Alzheimer’s disease (AD) is marked by cognitive dysfunction, a loss of memory and learning abilities which gradually contributes to neurodegenerative disease [1,2]. Although the exact cause of Alzheimer’s disease is unknown, it is widely accepted that excessive amyloid *β* protein deposits as well as abnormal neurofibrillary tangles, play a role in its pathogenesis [3]. In addition, various factors like neuroinflammation, oxidative stress, changes in the level of neurotrophic factors, and cholinergic dysfunction also lead to neuronal degeneration in AD [4,5,6].

Memory is linked with brain plasticity, which is further regulated by a plethora of factors, including BDNF, a neurotrophin that promotes neuronal survival and memory persistence [7,8]. BDNF is highly detected in the hippocampus and cortex, the portions of the brain that control learning, memory, and critical thinking [9,10]. The binding of BDNF to its receptor, tropomyosin-related kinase B (TrkB), initiates numerous signaling pathways, including phosphorylation of CREB followed by direct activation of CREB-dependent transcription and further gene expression of BDNF [11]. Further activation of extracellular signal-regulated kinase (ERK), a member of the mitogen-activated protein kinase family, can lead to improvements in cognitive abilities as it can activate CREB and further regulate numerous neuronal processes, including learning and memory formation [12,13]. These signaling molecules are also regulated by neurotransmitters and hormones like acetylcholine (ACh). Hippocampal tissues of AD patients and scopolamine-induced mouse models reportedly show down-regulation of BDNF expression [14,15]. Therefore, BDNF is a biomarker of cognitive function, and therapeutic approaches targeting the ERK-CREB-BDNF pathway can be an effective strategy in developing potential therapeutics for cognitive impairment [16,17].

An imbalance in the cholinergic system occurs with changes in the level of acetylcholine, a neurotransmitter in the brain that results from a reduction in numerous cognitive abilities. Aging and AD are associated with deficits of ACh due to hydrolysis by acetyl choline esterase at the synaptic site of the brain, therefore, targeting AChE activity is a potential therapeutic approach for memory-enhancing research or AD [18,19]. ACh esterase inhibitors such as donepezil, galantamine, and rivastigmine, are recommended for the treatment of cognitive dysfunction associated with AD, but their application is limited due to their resulting side effects [20,21,22]. This necessitates the development of safe and natural therapeutic products for the treatment of AD.

Medicinal herbs and plants have been studied for their potential in pharmaceutical and biological activities over the last several decades. In this context, researchers have elucidated a wide spectrum of pharmacological properties of EA, including antihyperglycemic, immune-modulating, anti-atherosclerotic, anti-tumor, and anti-inflammatory [23]. Several compounds, including flavonoids, terpenoids, steroids, lignans, cardenolides, phenolic acids, and alkaloids have been isolated and identified from EA leaves and twigs [24]. On the other hand, it is evident that intake of flavonoid-enriched foods remarkably improves neurogenerative disorders, including cognitive impairment, senescence process, and AD [25,26]. Woo et al. found that EA leaf extract enriched with rutin had strong antioxidant effects and improved memory acquisition via BDNF-induced Nrf2 activation [27]. More accumulated evidence suggests that different flavonoids, including naringenin, aromadendrin, and taxifolin in EA leaves and twigs also exhibit inhibitory effects on LPS-induced NO release in microglia [28].

Nevertheless, there is no report that has been done to investigate the therapeutic effects of EA twig extract. Hence, our main aim was not only to evaluate the ameliorating effects of EA twig extract on mice’s cognitive performance but also to determine the mechanism of their preventive effects in hippocampi and B35 cells against scopolamine-induced stress. Moreover, the protective effects of isolated flavonoids were also determined, either alone or in combinations.

## 2. Materials and Methods

### 2.1. Preparation of the Euonymus Alatus Twig Extract

The EA was collected in Asan, South Korea, in November 2019, and the plants were air-dried and stored at −20 °C before use for extraction. Two types of ethanol extracts; EA twig extract 1 and EA twig extract 2. The dried twigs of EA twigs (2 kg) were extracted with 70% ethanol (2 × 20 L) for 4 h (2×) at 80 °C. The extract was filtered through filter paper. The solvent was evaporated under reduced pressure to yield the concentrated twig extract 1 (174.0 g). Then, twig ethanol extract 1 (9 g) was suspended in 70% ethanol and fractionated with hexanes (4×). A portion of 70% ethanol was evaporated under reduced pressure to yield the concentrated twig extract 2 (6.7 g). Later, twig extract 1 and twig extract 2 were used for further analysis.

### 2.2. HPLC, ESI MS and NMR

Waters Alliance HPLC system (Waters, Houston, TX, USA) consisting of a binary pump, an online degasser and a diode array detector (DAD) was used to determine the contents of flavonoids. For chromatographic analysis, a Sunfire C18 (4.6 mm × 250 mm, 5 µm) column was used at room temperature. The mobile phase consisted of 0.08% TFA water (A) and acetonitrile (B) using a gradient program of 5–15% (B) in 0–5 min, 15–35% (B) in 5–40 min, 35–50% (B) in 40–55 min, 50–65% (B) in 55–60 min, 65–100% (B) in 60–70 min, and 100% (B) in 70–80 min. All the solutions were filtered through 0.22 μm to the HPLC analysis and monitored at 280 nm. The mass spectra were recorded in Waters micromass Z_Q_ by electronspray ionization (ESI) in the positive and negative mode. The parameters were as follows: capillary voltage: 2.50 KV; cone voltage: 40 V; extractor voltage: 3 V; RF lens voltage: 0.3 V; source temperature: 100 °C; desolvation temperature: 200 °C; desolvation gas flow rate: 350.0 L/h; cone gas flow rate: 50.0 L/h; scanning range: from 100 to 1000 amu. ^1^H NMR and ^13^C NMR spectra were recorded on varian VNS (600 MHz and 150 MHz, respectively) spectrometers in deuterated acetone.

### 2.3. Isolation of Flavonoids and Fragmentation of Compounds

**Figure 1:**
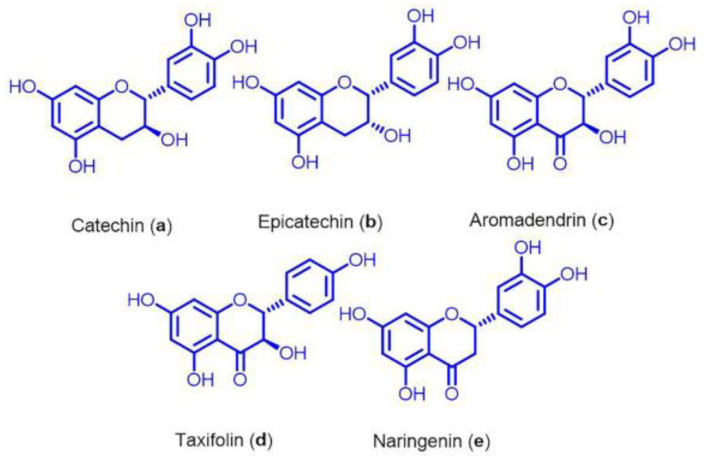
Isolated flavonoids; catechin (**a**), epicatechin (**b**), aromadendrin (**c**), taxifolin (**d**), and naringenin (**e**) from *Euonymus alatus* twig extract.

Twigs extract 1 (10 g) was suspended in deionized water (500 mL) and sequentially fractionated with hexanes (4 × 500 mL) and ethylacetate (2 × 500 mL). The portion of ethylacetate extract was first separated through silica gel column chromatography (reverse phase, silica C18) by using H_2_O: MeOH (95:5 to 50:50) to afford fraction 1 and fraction 2. Catechin (**a**)**,** and epicatechin (**b**) were isolated from fraction 1 through silica gel column chromatography (normal phase) using CHCl_3_: MeOH (95:5 to 90:10). Similarly, aromadendrin (**c**), taxifolin (**d**), and naringenin (**e**) were isolated by the normal phase silica gel column chromatography of fraction 2. The isolated flavonoids are shown in Figure 1. The isolated compounds were analyzed through HPLC, NMR, and ESI MS analysis [Supplementary Materials]. Similarly, three fractions were isolated from twigs extract 1 using preparative reversed phase-column chromatography using the solvent system H_2_O: MeOH (90:10–0:100) (SM–S7). The collected fractions were classified as fraction A (Fr. A), B (Fr. B), and C (Fr. C) on the basis of HPLC chromatogram (Figure S12.). Later, each fraction was analyzed for the in vitro assay.

### 2.4. Materials

The standard drug donepezil and scopolamine hydrobromide were purchased from Sigma-Aldrich Chemical Co, St Louis, MO, USA. DMEM, Fetal Bovine serum, Antimycin A and TryPLE express were acquired from Gibco (ThermoFisher Scientific, Waltham, MA, USA). Antibody directed against BDNF (bsm-52368R) was purchased from Bioss (Beijing, China), whereas antibodies of *p*-ERK (CST9102), ERK (CST9102), *p*-CREB (CST9198), and CREB (CST4820) were sourced from Cell Signaling Technology Inc (Beverly MA, USA). Antibodies against β-Actin (ab8226) were procured from Abcam (Cambridge, MA, USA).

### 2.5. Cell

B35, a rat neuroblastoma cell line was obtained from the American Type Culture (ATCC, CRL-2754). The cells were cultured in Dulbecco’s Modified Eagle’s Medium (DMEM) supplemented with 10% fetal bovine serum and 1% Antimycin A, at 37 °C in a humidified incubator under 5% CO_2_ atmosphere. The media were changed every 2 days and sub-cultured when they reached 80–90% confluency.

### 2.6. Drug Preparation

Extracts of EA twigs in the current study were prepared in the chemical research laboratory of Dongsung Cancer Center, Daegu, and stored at 4 °C in dark conditions. The doses of the EA twig extract and donepezil were selected on the basis of reports published in previous literature. EA twig extracts 1 and 2 were dissolved in 5% ethanol in normal saline while donepezil was dissolved in normal saline, and further these solutions were sonicated for 10 min and administered to the animals. Scopolamine was prepared fresh (2 mg/kg) in physiological saline and was protected from light by wrapping the tube with aluminum foil. For the in vivo experiments, EA twig extract was dissolved in DMSO to make the concentrated stock solutions of 50 and 100 mg/mL, which was then further diluted 1:1000 in media to give the desired concentration of 50 and 100 µg/mL. 

### 2.7. Animal

Male ICR mice aged 6 weeks and weighing 35 g were purchased from Orient Bio (Sungnam, Korea) and acclimated in a controlled animal house facility at the Dongsung Cancer Center, Daegu, for 7 days with an alternate light and dark cycle of 12 h each before behavioral experiments were carried out. Male mice were selected over female mice because of a benefit that was consistently noted in investigations using animal behavioral tests [29,30]. Each experimental group consisted of randomly grouped mice of the same weight. All the animal experiments were reviewed and carried out with the approval of the Animal Experimental Committee of the Dongsung Cancer Center under the protocol of IACUC #ds002106117-2. The experiments were carried out in compliance with the ARRIVE guidelines.

### 2.8. Animal Model for Cognitive Impairment

Korl:ICR mice (n = 40) were randomly divided into normal (n = 10) and scopolamine-induced (n = 30). We performed a power analysis to estimate the probability of detecting the optimal number of mice using a previously published study [31]. The normal group was not exposed to any experimental treatment, while the scopolamine-treated group was divided into three subgroups with 10 mice in each group as shown in Table 1. Animals were treated by oral gavage with saline, EA twig extracts 2 (300 mg/kg) and donepezil (5 mg/kg) once per day in groups 2, 3, and 4, respectively. The dosing was carried out for a period of 34 days, followed by scopolamine administration from the 28th day up to the end of the experimental period. 

### 2.9. Passive Avoidance Test (PAT)

Mice were challenged for passive avoidance testing on the 33rd and 34th days. Mice were injected intra-peritoneally (I.P) with normal saline or scopolamine 30 min after oral administration of EA twig extract 2 and donepezil in their respective groups. Behavioral tests were performed 30 min after scopolamine or vehicle treatment. PAT was conducted in identical illuminated and non-illuminated boxes (Jeong do BNP, Seoul, Rupublic of Korea) separated by a guillotine door. Before the mice were trained, a habituation trial was performed to adapt the animals to the apparatus. Individual animals were put into the apparatus and allowed to freely explore the non-illuminated box for 60 s before the light was turned on and the guillotine door was opened. The mice were allowed to enter the dark room for 30 s. This was performed on the 34th day without the drug and scopolamine treatments. In this test, the initial latency time of the entrance of the mice to the dark room from the time the door opened was recorded. On the 34th day, passive avoidance training was performed. For 10 s, each mouse was gently held with their bodies and placed in a non-illuminated room with the guillotine door closed. A white light was turned on, and the door was opened for the mouse to enter the dark room. Upon entering the entire body of the animal, the door was closed, and an electronic shock (0.5 mA, 3 s) was applied. The animals were subsequently returned to their respective cages. After 24 h, the animals were placed in the illuminated room and latency was recorded to check retention time. The record of latency for each mouse was determined within 300 s. After the behavioral testing, the brain was rapidly dissected to obtain hippocampal sections, which were immediately stored at −80 °C.

### 2.10. Western Blot Analysis

Mice were sacrificed by cervical dislocation after 1 h following drug and scopolamine administration. The brain was excised to isolate bilateral hippocampal regions and was stored at −80 °C for Western blot analysis. The protein of hippocampal tissues (20 mg) was extracted by using 400 µL of RIPA buffer (0.15M Sodium chloride, 1% Triton X-100, 1% Sodium deoxycholate, 0.1% SDS, 50 mM Tris adjusted to pH 7.5) with 1x proteases and a phosphatase inhibitor (Gen-DEPOT, USA) and were homogenized by tissue homogenizer. 

B35 cells were seeded at the density of 4 × 10^5^ cells/well and pre-treated with or without EA twig 1 and 2 extract (50 and 100 µg/mL) and donephezil (1 and 5 µM) prior to the treatment with scopolamine (2 mM) for 24 h. Then, the culture medium was removed and protein was isolated by lysing the cell with RIPA lysis buffer containing 1x proteases and phosphatase inhibitor (Gen-DEPOT, USA). Following that, the cell or tissue lysate were then centrifuged for 15 min at 15,000 rpm, and the protein content in the supernatant was determined using Bio-Rad, protein assay reagent. The expression of BDNF, ERK/ *p*-ERK, and CREB/*p*-CREB proteins in the hippocampus was determined by Western blot. The proteins from homogenates were separated by 10% polyacrylamide gel electrophoresis and transferred to polyvinylidene fluoride (PVDF) membranes. After blocking in 5% skim milk, the membranes were probed with primary antibodies overnight at 4 °C. The membranes were washed and incubated for 2 h at room temperature with an anti-rabbit antibody. The membrane was further visualized using an enhanced chemiluminescent reagent (Pierce Biotechnology, Rockford, IL, USA), on a luminescent image analyzer (ImageQuant Las 500, Cytiva, Tokyo, Japan)

### 2.11. Cell Viability Test

B35 cells (2 × 10^5^/mL) were placed in 96-well plates. Cells were pretreated with or without 100, 200, 300 and 400 µg/mL of EA twig extract 1 or 20, 40, 60, 80, 100, 120, 140, and 160 µg/mL of EA twig extract 2 in DMSO for 72 h. The colorimetric 3-(4,5-dimethylthiazol-2-yl)-2,5-diphenyltetrazolium bromide (MTT) staining method was used to assess cell viability. After treatments, cells were incubated with 0.5 mg/mL MTT at 37 °C in the dark for 3 h under 5% CO_2_ humidified incubator, and then 200 µL of DMSO was added to dissolve reduced MTT crystals. Absorbance was measured with a microplate reader (Multiscan GO, Thermo Scientific, Vantaa, Finland) at 570 nm filters.

### 2.12. Acetyl Choline Esterase Activity Assay

Briefly, B35 cells were seeded at 2 × 10^4^ cells in a 96-well microplate in triplicates and treated with different concentrations of EA twig extracts 1 and 2 (50 and 100 µg/mL) and donepezil (20.8 µg/mL) followed by treatment with 2 mM of scopolamine for 24 h. Control was prepared without the treatment with extract and scopolamine. Lysates were centrifuged at 1500 rpm for 5 min, and the supernatants were used for AChE activity. The AChE activity was measured by using the Abcam acetylcholinesterase assay kit (ab138871) according to the manufacturer’s protocol. Absorbance was measured in a microplate enzyme-linked immunosorbent assay (ELISA) reader (Multiscan GO, Thermo Scientific, Vantaa, Finland) at 410 nm. The data obtained were compared to the standard curve of AChE.

### 2.13. Statistical Analysis

All data were expressed as mean ± standard deviation (SD). Data were analyzed by one-way ANOVA with Tukey’s post hoc test using the GraphPad Prism software (version 5.01, Inc., 2007, San Diego, CA, USA). Data with probability values of less than 0.05 were considered statistically significant. In addition, Student’s t-tests were also used to confirm the significance of each measurement value.

## 3. Results

### 3.1. Effect of EA Twig Extract on Viability of B35 Neuroblastoma Cell Line

**Figure 2:**
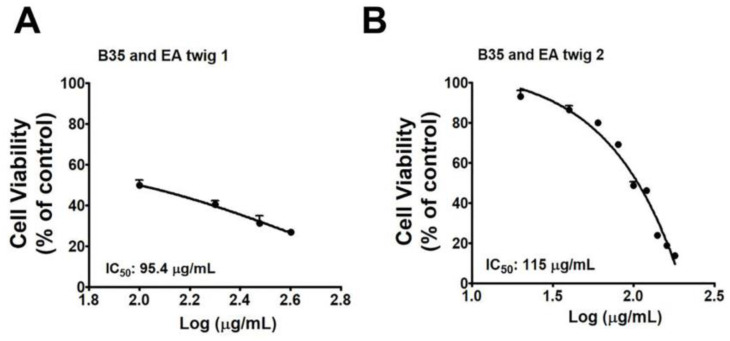
IC50 values for EA twig extracts in cytotoxicity assays with the B35 cell line. (**A**) Cell viability of the EA twig extract 1 and (**B**) EA twig extract 2 accessed by MTT assay in B35 cell line. Values are representative of mean ± SD obtained from at least three independent experiments.

To investigate whether or not ethanol extracts of EA twig extracts 1 and 2 have toxic activities on normal neuroblastoma cells, different concentrations of the twig extract 1 (100, 200, 300, 400, and 500 µg/mL) and twig extract 2 (20, 40, 60, 80, 100, 120, 140, and 160 µg/mL) were treated with cells and the number of viable cells was measured by MTT assay (Figure 2A,B). Two different neuroblastoma cell lines, SH-SY5Y (human neuroblastoma cell line) and B35 (a rat neuroblastoma cell line) were employed. In contrast to B35 cells, twig extracts were more toxic to the SH-SY5Y cells (data not shown). B35 cell lines were consequently chosen for further studies. Twig extracts 1 and 2 showed a dose-dependent cytotoxic effect in the rat neuroblastoma cell line, B35. The result showed that the amounts of EA twig extracts 1 and 2 needed for 50% cytotoxicity of B35 cells were 95.4 and 115 µg/mL, respectively. Thus, the EA twig extract 2 showed better cell viability compared with the EA twig extract 1. 

### 3.2. Effect of EA Twig Extract on the Scopolamine-Induced B35, Rat Neuroblastoma Cells

**Figure 3:**
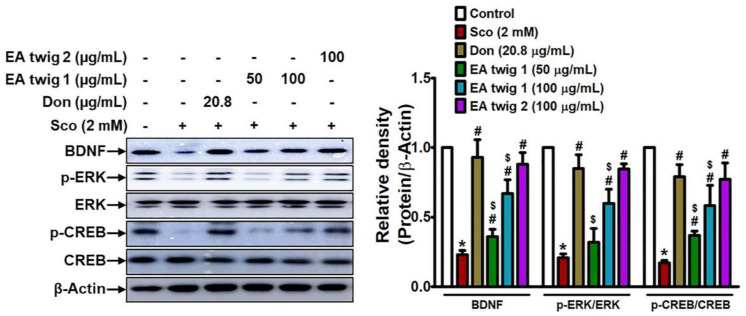
Effects of EA twig extracts 1 and 2 on BDNF expression and phosphorylation of ERK and CREB in scopolamine-treated B35 cells. BDNF, *p*-ERK, and *p*-CREB expressions were increased in B35 cells with increasing concentrations of EA twig extracts 1 and 2, respectively. The data are expressed as relative density of protein expression vs. β-Actin. Values are shown as mean ± SD obtained from at least three independent experiments and were analyzed by one-way ANOVA with Tukey’s post hoc test. * *p* < 0.05, compared with the vehicle-treated control group. ^#^ *p* < 0.05, compared with the scopolamine-treated group. ^$^ *p* < 0.05, compared with the donepezil-treated group.

Scopolamine concentrations of 2 mM were evaluated against EA twig extracts 1 and 2 for 24 h as part of the evaluation of their neuroprotective effects. 2 mM dose of scopolamine statistically reduced B35 cell viability (SM–S8, Figure S13A). Scopolamine-mediated toxicity was slightly decreased by extract incubation at 25–50 µg/mL concentrations of EA twig extract 2 while exposure to more than 50 µg/mL dose showed cell toxicity (Figure S13B). Compared to EA twig extract 2, EA twig extracts 1 displayed lesser neuroprotection (Figure S13C). To validate the role and the molecular mechanisms underlying the treatment of EA-twig extract in preventing scopolamine-induced B35 cells, a decrease in the expression of BDNF and related proteins was measured by Western blot analysis. As shown in Figure 3, scopolamine treatment decreased the level of BDNF expression in accordance with decreased ERK and CREB phosphorylation. However, EA twig extract 1 gradually restored the scopolamine-down regulated molecular signaling pathway in a concentration-dependent manner and showed the highest restoration at a concentration of 100 μg/mL. EA twig extract 2 (100 μg/mL) showed better effects in B35 cells than twig extract 1 but to a lesser degree than donepezil (20.8 µg/mL), a standard drug. Unlike EA twig extract 1, EA twig extract 2 is a hexane-washed extract from which chlorophyll related compounds have been eliminated. EA twig extract 2 thus exhibited the best efficacy by increasing the aforementioned memory-associated proteins in B35 cells. 

### 3.3. In-Vitro AChE Inhibitory Activity of EA Twig Extract

**Figure 4:**
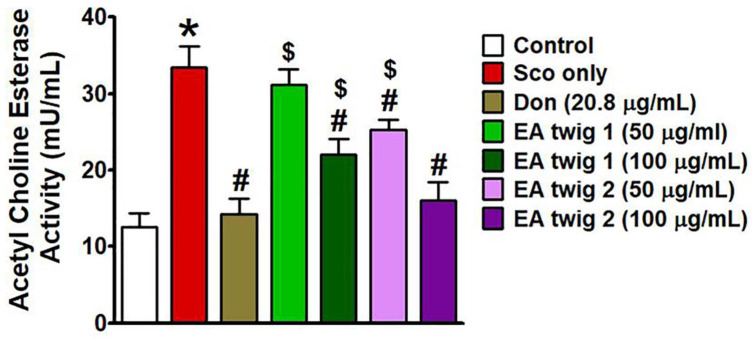
Effects of EA twig extracts 1 and 2 on AChE activity in scopolamine-induced B35 cells. Representative data are shown as mean ± SD and were analyzed by one-way ANOVA with Tukey’s post hoc test. * *p* < 0.05, compared with the vehicle-treated control group. ^#^ *p* < 0.05, compared with the scopolamine-treated group. ^$^ *p* < 0.05, compared with the donepezil-treated group.

Many studies have shown that a reduced acetyl choline level is one of the hallmarks of neuronal cells in AD [32], therefore the activity of EA twig extracts was assessed by determining acetyl choline esterase activity in scopolamine-induced B35 cells. AChE activity was higher in the scopolamine-induced group than in the control group. However, co-treatment with both EA twig extracts 1 and 2 resulted in a concentration-dependent increase in the percentage inhibition of the AChE enzyme when tested in the concentration range of 50 to 100 μg/mL (Figure 4). The results were compared to the standard drug donepezil (20.8 µg/mL). From the results of AChE activity assay we can conclude that EA twig-extract 2 (100 μg/mL) was a potent inhibitor of AChE in comparison to that of EA twig-extract 1 (100 μg/mL), as it showed two times better inhibition of AChE activity increased by scopolamine. 

### 3.4. Effects of Different Flavonoids Isolated from EA Twig Extract on Scopolamine-Induced B35 Cells

Since flavonoids are the major constituents of EA twig and their pharmacological effects can be accredited to their antioxidant effects, we thought that flavonoids might interact in CREB-BDNF pathway. Therefore, we evaluated the potential of each isolated flavonoids; catechin (**a**), epicatechin (**b**), aromadendrin (**c**), taxifolin (**d**), and naringenin (**e**) from EA twig on scopolamine-induced B35 cells. Scopolamine interventions reduced the expression of BDNF, p-ERK, and p-CREB, but EA twig extract and each of the flavonoids significantly increased these expressions in comparison to the control. Test compounds were dissolved in DMSO at concentrations of 100 μg/mL. 

**Figure 5:**
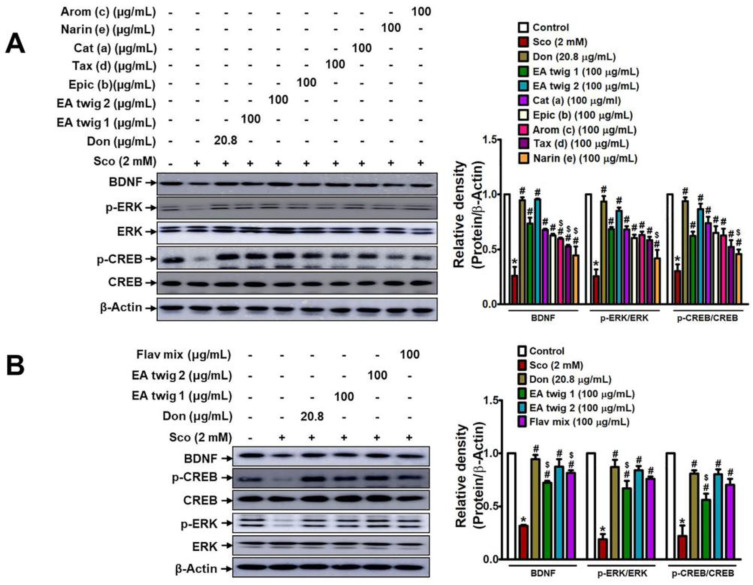
Flavonoids from EA twig extract reversed scopolamine-induced down-regulation of BDNF, phosphorylated ERK, and CREB in B35 cells. B35 cells were pretreated with Donepezil (20.8 µg/mL), EA twig extract 1, 2, and (**A**) flavonoids, i.e catachin (**a**), epicatachin (**b**), aromadendrin (**c**), taxifolin (**d**), and naringenine (**e**) in 100 µg/mL, respectively, (**B**) flavonoid mixtures of 100 μg/mL (prepared by mixing **a**–**d** in equal ratio) for 1 h followed by treatment with scopolamine for 24 h. The cells were lysed an-d Western blot analysis was performed to analyze the levels of BDNF, *p*-ERK, and *p*-CREB. Protein expression levels were normalized to those of β-actin. Representative datas are shown as mean ± SD from at least three independent experiments and were analyzed by one-way ANOVA with Tukey’s post hoc test. * *p* < 0.05, compared with vehicle-treated control group. ^#^ *p* < 0.05, compared with the scopolamine-treated group. ^$^ *p* < 0.05, compared with donepezil-treated group.

Our findings revealed that the degree to which isolated flavonoids enhanced the production of BDNF, p-ERK, and p-CREB varied; however, flavonoids, catechin (**a**) and epicatechin (**b**) showed the best effects as they most effectively decreased scopolamine-induced effects, followed by flavonoids, aromadendrin (**c**), taxifolin (**d**)**,** and naringenin (**e**) (Figure 5A). Similarly, among the Fr. A, B, and C, Fr. A of the twig extract demonstrated a substantial effect in reversing the scopolamine-induced effects (SM–S9, Figure S14) and was found to be rich in flavonoids particularly **a**–**d** where **a** was the major group of flavonoids enriched in it. In addition, a combination of flavonoids **a**, **b**, **c,** and **d** exhibited stronger effects on the restoration of BDNF, p-ERK, and p-CREB expressions by 4.5 folds in scopolamine-induced B35 cells. Individual flavonoids only increased these expressions by 2–3 folds (Figure 5B).

### 3.5. Mixture Effect of EA Twig Extract 2 and Catechin (a) on BDNF and Its Downstream Protein Expression in B35 Cells

Further, previous literature has shown that the consumption of catechin and epicatechin-rich foods can improve memory in experimental animals [33]. Additionally, it was discovered that catechin (**a**), a key flavonoid in EA twig extract, decreased memory deficits induced by scopolamine via activating the CREB–BDNF pathway. As a result, the impact of flavonoid **a** on BDNF and p-CREB-protein levels in the extract was also investigated. Further, to determine the interactions of flavonoid **a** with EA twig extracts, mixtures of flavonoid **a** with EA twig extract 1 or EA twig extract 2 were made to evaluate their additive properties. Mixtures of EA twig extracts 1 or 2 with flavonoid **a** (7:3 *v*/*v*) exhibited additive interactions, enhancing the expressions of BDNF and its downstream signaling molecule p-ERK and p-CREB, which had been reduced by scopolamine. However, the combined effect of EA twig 2 extract and flavonoid **a** showed more pronounced effects in B35 cells than single treatments of EA twig extract 1, 2, or flavonoid **a** (SM–S10, Figure S15). 

### 3.6. EA Twig Extract 2 Improves the Scopolamine-Induced Memory Impairments in ICR Mice

**Figure 6:**
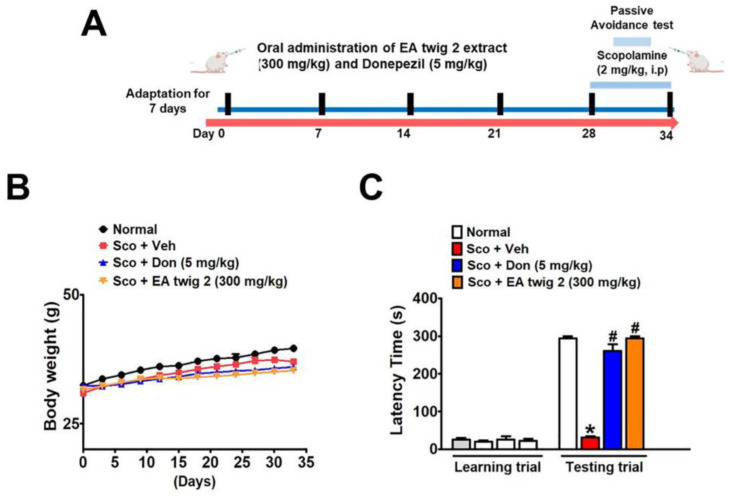
The effects of EA twig extract 2 on scopolamine-induced memory impairment in the passive avoidance test. (**A**) Timeline for the experimental procedure of drug treatment and the passive avoidance test. For 34 days, mice in different groups were given an equivalent amounts of saline; donepezil (5 mg/kg) and EA twig 2 (300 mg/kg), respectively. Memory deficits were induced by an intraperitoneal scopolamine injection (2 mg/kg) 30 min before the trial. The control group was injected with saline. (**B**) Mouse body weights. (**C**) Latency time for training and the passive avoidance test. The data are expressed as mean ± SD, n = 5 and were analyzed by one-way ANOVA with Tukey’s post hoc test. * *p* < 0.05, compared with the vehicle-treated control group. ^#^ *p* < 0.05, compared with the scopolamine-treated group.

Next, we used a scopolamine-induced cognitive impairment model in ICR mice to determine the memory-ameliorating effects of EA twig extract 2. The experimental schedule of drug and scopolamine administration for animal behavior tests is shown in Figure 6A. Since EA twig extract 2 showed promising activity in vitro, we selected twig extract 2 for the in vivo mouse model. The effect of EA twig extract 2 enhances the restoration of memory impairments as performed by PAT. The mean step-through latency of scopolamine-treated mice (2 mg/kg, i.p.) was significantly shorter than that of vehicle-treated control mice (* *p* < 0.05) and was significantly elongated by EA twig extract 2 (300 mg/kg, p.o.) and donepezil (5 mg/kg, p.o.) treatment after 28 days (Figure 6B). It was also revealed that this learning improvement activity exhibited by EA twig extract 2 was almost comparable to the standard drug, donepezil. The correlation between this result and the in vitro data showed good agreement, suggesting EA twig extract 2 had the best cognitive-enhancing effect. No significant difference was observed in the weight of animals during the course of the study (Figure 6C). 

### 3.7. EA Twig Extract 2 Rescues ICR Mice from Scopolamine-Induced Deficits by Restoring BDNF Signalling

**Figure 7:**
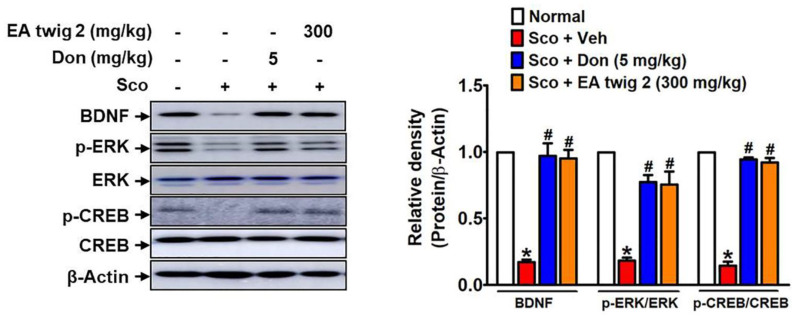
EA twig extract 2 reverses down-regulated *p*-ERK, *p*-CREB, and BDNF expressions in the mouse hippocampus. Protein expressions of *p*-ERK, *p*-CREB and BDNF in the hippocampus of scopolamine-treated mice by immunoblot analysis. The bar represents mean ± SD from brain extracted from three different mice and were analyzed by one-way ANOVA with Tukey’s post hoc test. * *p* < 0.05, compared with the vehicle-treated control group. ^#^ *p* < 0.05, compared with the scopolamine-treated group.

To confirm the preventive effect of EA twig extract 2 in scopolamine-induced cognitive deficit hippocampal tissues (Figure 7), Western blot analysis of BDNF, *p*-ERK, and *p*-CREB was conducted. Results demonstrated that the scopolamine-treated group exhibited decreased BDNF, *p*-ERK, and *p*-CREB expression compared to the normal control mice. Administration of EA twig extract 2 (300 mg/kg) recovered the scopolamine-decreased BDNF and its down-stream molecules (ERK and CREB) in the hippocampus. Furthermore, treatment with donepezil (5 mg/kg) also showed a similar attenuating effect.

## 4. Discussion

EA has been used as a traditional medicine in East Asia for the treatment of various diseases including neurodegenerative diseases [34]. There have recently been reports that the leaves of EA have antioxidant properties and can help with scopolamine-induced amnesia [7]. In this regard, the major chemical profiles of the leaves of EA are similar to those of the twigs of EA [24]. Therefore, we decided to scientifically test whether EA twigs can attenuate cholinergic blockade-induced memory impairment. In the current study, we investigated the potential neuroprotective effects of two different EA twig extracts 1 and 2, and their active flavonoids in both an in vitro and in vivo model of scopolamine-induced deficits.

Since it is well known that neuroblastoma cells are excellent for evaluating neuroprotective medications, we chose two neuroblastoma cell lines, SH-SY5Y and B35 cells for this investigation over neuronal cells [35]. However, due to the high cytotoxicity of EA twig extract against SH-SY5Y cells, we used an in vitro neuronal-derived cell model, B35 neuroblastoma cells to determine the direct effects of EA twig extract 1 and 2 on cholinergic and BDNF/CREB signaling. At 100 μg/mL concentration, twig extracts 1 was found to be cytotoxic while twig extract 2 exhibited slight toxicity against rat neuroblastoma cells. This difference might be due to different extraction procedures and differences in the phytochemical composition of the extracts. Moreover, studies have shown that plant extracts have pharmacologically active substances contained in them but chlorophyll-containing components in the extract can interfere with the analytical properties of the natural products [36]. Thus, it can be inferred that removal of the chlorophyll bearing components from twig extract can help to improve its efficacy. EA twig extract 1 was not appropriate, therefore EA twig extract 2 was selected for further in vivo and in vitro analysis.

The cholinergic system plays a pivotal role in learning and memory storage and shows degenerative patterns in AD patients [37,38]. A number of ChE inhibitors used to treat AD, have various dose-associated side effects [39]. Therefore, many nutraceutical compounds like herbs or herb-derived, nutrient-derived substances are encouraged to develop as cognitive enhancing drugs. Likewise, herbs like EA and its twig extract are rich in flavonoids like catechin, epicatechin, rutin, and quercetin, and from other reports it is revealed that these compounds have the ability to antagonize AChE by enhancing the ACh concentrations in the synaptic cleft of cholinergic neurons [24,40]. Studies have also shown that most of the AChE inhibitory activity of these flavonoids is imparted by the positions and arrangement of the hydroxyl (OH) group at rings A and B, and due to the unsaturation of ring C in phenyl groups [40]. In the present study, we measured the AChE inhibitory activity of EA twig extract by using scopolamine-induced B35 cells. The effective flavonoids present in EA twig extracts 1 and 2 inhibited scopolamine-enhanced AChE activity in B35 cells in a dose-dependent manner. This indicates that inhibition of AChE activity must be one mechanism by which EA twig extracts improve cognitive impairments.

The present investigation demonstrated that extracts of EA twig 2 (300 mg/kg) significantly ameliorated cognitive impairment induced by scopolamine in a mouse model (*p* < 0.05). To the best of our knowledge, this study is the first to demonstrate that EA twig extracts exert memory-improving effects. In this study, the administration of scopolamine (2 mg/kg) for a week was able to induce cognitive impairment in mice, as shown by the passive avoidance test. While pre-treatment with EA twig extract (300 mg/kg, i.p) for 34 days greatly decreased the scopolamine-induced latency in mice in contrast to those that only received scopolamine. Furthermore, as demonstrated by PAT, EA twig extract 2 (300 mg/kg) and donepezil (5 mg/kg) were equally beneficial, and these findings fit with the AChE inhibitory activity. However, donepezil (20.8 μg/mL) had more effective action than EA twig 2 extracts (100 μg/mL) in scopolamine-induced B35 cells. Based on these results, it can be concluded that EA twig extract 2 treatments greatly enhanced learning and memory in scopolamine-induced mice.

Accordingly, we elucidated the molecular mechanism underlying the therapeutic activity of EA twig extract to understand how memory impairment induced by scopolamine is improved. In AD, there is growing evidence that alterations in the levels of BDNF play a role in cognitive dysfunction [41]. In this study, our results revealed that scopolamine induction in ICR mice decreased BDNF protein expression in hippocampal mouse tissues. In the present study, we also used scopolamine-treated B35 cells to mimic this process and to evaluate the protective effect of EA twig extract. Scopolamine is a muscarinic receptor antagonist that blocks cholinergic transmission in rodents, causing short-term memory loss similar to that seen in Alzheimer’s [42,43]. Furthermore, activation of downstream signaling molecules like ERK and CREB signaling molecules is also associated with neurotransmitters like acetylcholine and activation of their receptors [44]. In view of these, scopolamine-induced memory deficits lead to decreased expressions of hippocampal BDNF and CREB, which further interfere with hippocampal-dependent memory function, and these effects observed in our study were consistent with those previously reported [45,46]. The mitogen-activated protein kinase (MAPK) signaling pathway is implicated in learning and memory through interaction with the cholinergic system. ERK, a MAPK kinase, activates CREB, a downstream signaling molecule that regulates cellular processes for synaptic activity and neuronal survival [47]. It is worth noting that the enhanced BDNF secretion was paralleled by increased phosphorylation levels of ERK and CREB, as demonstrated in the scopolamine-induced hippocampal tissues of mice. Our data also demonstrated that the treatment with EA twig extracts 1 and 2 at a concentration of 100 μg/mL reversed the down-regulation of a CREB-BDNF signaling pathway in scopolamine-treated B35 cells. Indeed, EA twig extract 2 was shown to have a more potent ability to enhance BDNF expressions compared to EA twig extract 1. 

The phytochemical screening of crude EA twig extracts revealed the presence of different flavonoids, of which five were isolated. Therefore, to determine the active constituent(s) of EA, we tested the isolated flavonoids against scopolamine-induced B35 cells. All the compounds in this study, at 100 μg/mL concentration were able to suppress the scopolamine-induced changes in B35 cells. However, flavonoid **a** was the most potent and abundant flavonoid in EA twig extract. At 100 μg/mL, it restored BDNF and phosphorylation of ERK and CREB, but at a lower level than EA twig extract 2 itself. Several studies have revealed the recovery of flavonoids like catechin, and dihydromyricetin in high amounts from EA extract [48]. In the present study, we also obtained the highest level of **a** from EA twig extract, which demonstrated strong protective potential in vitro. Subsequently, high temperature processed-green tea extract containing catechin promoted neuronal differentiation in SH-SY5Y cells and attenuated memory deficits in mice, which supports our findings [49]. Several previous studies [50,51] have linked catechin and its derivatives’ protective action to their strong antioxidant and chelating effects. Secondly, catechin’s ability to penetrate the blood–brain barrier and brain to achieve significant levels helps in exerting neuroprotective action [52]. Other flavonoids like **b**, **c,** and **d** also showed good effects, while **e** showed the least effectiveness in the current study. Additionally, growing evidence from previous pharmacological studies has indicated similar neuroprotective findings for these flavonoids [53,54]. Among the fractions A, B, and C isolated from EA twig extract, fraction A generated a significant increase in BDNF, *p*-CREB, and *p*-ERK expression in B35 cells with scopolamine-inductions. This may be due to the maximum contents of flavonoids **a**, **b**, **c**, and **d** and their therapeutic effect. However, Fr. B and C displayed less effect, which might be attributed to the omission of the very polar compounds.

Further, we also tested the mixtures of **a**, **b**, **c**, and **d** (1:1:1:1) at a 100 μg/mL concentration to determine their effects on scopolamine-induced neuroblastoma cells. The mixture of flavonoids tested in scopolamine-treated B35 cells reveals the possibility of synergistic effects in increasing the BDNF/*p*-ERK/*p*-CREB expression levels. However, flavonoids alone did not show better effects relative to the EA twig extract. Therefore, the total effect of EA twig extracts might be attributed to the synergistic effects of the flavonoids present in them. Similarly, the results showed that **a** alone had less protective effects in B35 cells, but when combined with either EA twig extract 1 or 2, it had the best effects, implying that combining **a** with EA twig extract 1 or 2 can provide additive protection against scopolamine. This suggests that the addition of flavonoid a to the own composition of EA twig extract could increase the protective effect of the twig extract, therefore, such supplemented EA twig extracts can be more valuable in enhancing neuroprotective properties. In accordance with our results, previous reports have shown that extracts containing a mixture of flavonoid a may be more effective against AD than the individual flavanoid **a** [32]. 

## 5. Conclusions

Collectively, our study shows that twig extract of EA, a traditional medicine, resulted in the activation of the ERK-CREB-BDNF signaling cascade in the hippocampus and neuroblastoma cells, which may be the potential mechanism for the improved cholinergic function and, in turn, memory improving effects in scopolamine-injected mice. The concurrent biological modifications were attributed to the protective effects of flavonoids contained in EA twig extracts 1 and 2. We found that EA twig extract had anti-amnesic properties in scopolamine-induced neuroblastoma cells and a mouse model of memory impairment. Our data demonstrated that EA twig extract 2 ameliorated cognitive deficits measured by the passive avoidance test. EA twig extract 2 treatment reduced acetylcholine esterase activity, thus elevating acetylcholine levels in scopolamine-treated B35 cells. To the best of our knowledge, this is the first report highlighting the potential of EA twig extract to increase BDNF/*p*-ERK/*p*-CREB protein expression in vitro and in vivo studies. In addition, catechin (**a**) was identified as a major flavonoid in EA twig extract which restored the activation of the CREB–BDNF pathway to the fullest degree. The results indicate that the underlying mechanism of learning and memory improvement may involve modulations of the cholinergic system and the activation of BDNF/*p*-ERK/*p*-CREB signaling. Further understanding of the isolated flavonoids from twig extracts for blood–brain barrier permeation can provide more insights into their effects on the brain. These findings suggest that EA twig extract 2 or catechin supplemented with EA twig extract could be used as an effective dietary supplement to relieve daily stress related to learning and memory deficits and to achieve the goal of AD treatment in conjunction with other neurodegenerative disease treatments. 

## Figures and Tables

**Table 1:** Groups of mice in the scopolamine-induced cognitive model.

Group 1	Normal Mice
Group 2	Scopolamine (2 mg/kg, i.p)
Group 3	Scopolamine (2 mg/kg, i.p) + Donepezil (5 mg/kg, p.o)
Group 4	Scopolamine (2 mg/kg, i.p) + EA twig 2 extract (300 mg/kg, p.o)

i.p: Intraperitoneal p.o: Per os.

## Data Availability

All the data are contained within the manuscript and Supplementary Materials.

## Supplementary Materials

The following supporting information can be downloaded at: https://www.mdpi.com/article/10.3390/nu15010128/s1, Section S1: General information; Section S2: Characterization data of isolated compounds; Section S3: References; Section S4: Copies of ^1^H and ^13^C NMR spectra of isolated compounds; Section S5: Copies of ESI mass of isolated compounds; Section S6: HPLC analysis of EA twig extracts; Section S7: Prep. column chromatography of compounds; Section S8: Neuroprotective effectiveness of EA twig extract 1 and 2; Section S9: Effect of EA twig extract and its fraction on B35 cell; Section S10: Figure of mixture effect of EA twig extract 2 and catechin (a) on BDNF and its downstream protein expression in B35 cells; Section S11: Set 2 and 3 of Figures, include Figures S16–S18. Refs [55,56,57,58,59,60,61] cited in Supplementary Materials.

## References

[1] Wu S., Liu X., Jiang R., Yan X., Ling Z. (2021). Roles and Mechanisms of Gut Microbiota in Patients with Alzheimer’s Disease. Front. Aging Neurosci..

[2] Pugazhenthi S. (2017). Metabolic syndrome and the cellular phase of Alzheimer’s disease. Prog. Mol. Biol. Transl. Sci..

[3] Tillement L., Lecanu L., Papadopoulos V. (2011). Alzheimer’s disease: Effects of β-amyloid on mitochondria. Mitochondrion.

[4] Oh S.-Y., Jang M.J., Choi Y.-H., Hwang H., Rhim H., Lee B., Choi C.W., Kim M.S. (2021). Central administration of afzelin extracted from Ribes fasciculatum improves cognitive and memory function in a mouse model of dementia. Sci. Rep..

[5] Manoharan S., Guillemin G.J., Abiramasundari R.S., Essa M.M., Akbar M., Akbar M.D. (2016). The role of reactive oxygen species in the pathogenesis of Alzheimer’s disease, Parkinson’s disease, and Huntington’s disease: A mini review. Oxid. Med. Cell. Longev..

[6] Sampaio T.B., Savall A.S., Gutierrez M.E.Z., Pinton S. (2017). Neurotrophic factors in Alzheimer’s and Parkinson’s diseases: Implications for pathogenesis and therapy. Neural. Regen. Res..

[7] Woo N., Lu B. (2009). BDNF in synaptic plasticity and memory. Intracellular Communication in the Nervous System.

[8] Nieto R., Kukuljan M., Silva H. (2013). BDNF and schizophrenia: From neurodevelopment to neuronal plasticity, learning, and memory. Front. Psychiatry.

[9] Miranda M., Morici J.F., Zanoni M.B., Bekinschtein P. (2019). Brain-derived neurotrophic factor: A key molecule for memory in the healthy and the pathological brain. Front. Cell. Neurosci..

[10] Tapia-Arancibia L., Rage F., Givalois L., Arancibia S. (2004). Physiology of BDNF: Focus on hypothalamic function. Front. Neuroendocrinol..

[11] González-Gutiérrez A., Lazo O.M., Bronfman F.C. (2020). The Rab5–Rab11 Endosomal Pathway is Required for BDNF-Induced CREB Transcriptional Regulation in Hippocampal Neurons. J. Neurosci..

[12] Xue W., Wang W., Gong T., Zhang H., Tao W., Xue L., Sun Y., Wang F., Chen G. (2016). PKA-CREB-BDNF signaling regulated long lasting antidepressant activities of Yueju but not ketamine. Sci. Rep..

[13] Shi M., Ding J., Li L., Bai H., Li X., Lan L., Fan H., Gao L. (2021). Effects of Ketamine on Learning and Memory in the Hippocampus of Rats through ERK, CREB, and Arc. Brain Sci..

[14] Rosa E., Mahendram S., Ke Y.D., Ittner L.M., Ginsberg S.D., Fahnestock M. (2016). Tau downregulates BDNF expression in animal and cellular models of Alzheimer’s disease. Neurobiol. Aging.

[15] Choi J.M., Lee S.I., Cho E.J. (2020). Effect of Vigna angularis on High-Fat Diet-Induced Memory and Cognitive Impairments. J. Med. Food.

[16] Ayabe T., Ohya R., Taniguchi Y., Shindo K., Kondo K., Ano Y. (2018). Matured hop-derived bitter components in beer improve hippocampus-dependent memory through activation of the vagus nerve. Sci. Rep..

[17] Barak S., Weiner I. (2009). Towards an animal model of an antipsychotic drug-resistant cognitive impairment in schizophrenia: Scopolamine induces abnormally persistent latent inhibition, which can be reversed by cognitive enhancers but not by antipsychotic drugs. Int. J. Neuropsychopharmacol..

[18] Ferreira-Vieira T.H., Guimaraes I.M., Silva F.R., Ribeiro F.M. (2016). Alzheimer’s disease: Targeting the Cholinergic System. Curr. Neuropharmacol..

[19] Colovic M.B., Krstic D.Z., Lazarevic-Pasti T.D., Bondzic A.M., Vasic V.M. (2013). Acetylcholinesterase inhibitors: Pharmacology and toxicology. Curr. Neuropharmacol..

[20] Gualtieri F., Manetti D., Romanelli M., Ghelardini C. (2002). Design and study of piracetam-like nootropics, controversial members of the problematic class of cognition-enhancing drugs. Curr. Pharm. Des..

[21] Dwivedi P., Singh R., Malik M.T., Jawaid T. (2012). A traditional approach to herbal nootropic agents: An overview. Int. J. Pharm. Sci. Res..

[22] Takai K., Enomoto T. (2018). Discovery and development of muscarinic acetylcholine M4 activators as promising therapeutic agents for CNS diseases. Chem. Pharm. Bull..

[23] Oh B.K., Mun J., Seo H.W., Ryu S.Y., Kim Y.S., Lee B.H., Oh K.-S. (2011). Euonymus alatus extract attenuates LPS-induced NF-κB activation via IKKβ inhibition in RAW 264.7 cells. J. Ethnopharmacol..

[24] Zhai X., Lenon G.B., Xue C.C., Li C.-G. (2016). Euonymus alatus: A review on its phytochemistry and antidiabetic activity. Evid.-Based Complement. Altern. Med..

[25] Vauzour D., Camprubi-Robles M., Miquel-Kergoat S., Andres-Lacueva C., Bánáti D., Barberger-Gateau P., Bowman G.L., Caberlotto L., Clarke R., Hogervorst E. (2017). Nutrition for the ageing brain: Towards evidence for an optimal diet. Ageing Res. Rev..

[26] Williams R.J., Spencer J.P. (2012). Flavonoids, cognition, and dementia: Actions, mechanisms, and potential therapeutic utility for Alzheimer disease. Free Radic. Biol. Med..

[27] Woo Y., Lim J.S., Oh J., Lee J.S., Kim J.-S. (2020). Neuroprotective effects of euonymus alatus extract on scopolamine-induced memory deficits in mice. Antioxidants.

[28] Jeong E.J., Yang H., Kim S.H., Kang S.Y., Sung S.H., Kim Y.C. (2011). Inhibitory constituents of Euonymus alatus leaves and twigs on nitric oxide production in BV2 microglia cells. Food Chem. Toxicol..

[29] Astur R.S., Ortiz M.L., Sutherland R.J. (1998). A characterization of performance by men and women in a virtual Morris water task:: A large and reliable sex difference. Behav. Brain Res..

[30] Newhouse P., Newhouse C., Astur R.S. (2007). Sex differences in visual-spatial learning using a virtual water maze in pre-pubertal children. Behav. Brain Res..

[31] Sen S., Satagopan J.M., Broman K.W., Churchill G.A. (2007). R/qtlDesign: Inbred line cross experimental design. Mamm. Genome.

[32] Suthprasertporn N., Mingchinda N., Fukunaga K., Thangnipon W. (2020). Neuroprotection of SAK3 on scopolamine-induced cholinergic dysfunction in human neuroblastoma SH-SY5Y cells. Cytotechnology.

[33] Heo H.J., Lee C.Y. (2005). Epicatechin and catechin in cocoa inhibit amyloid β protein induced apoptosis. J. Agric. Food Chem..

[34] Bhat I.U.H., Bhat R. (2021). Quercetin: A bioactive compound imparting cardiovascular and neuroprotective benefits: Scope for exploring fresh produce, their wastes, and by-products. Biology.

[35] Peruche B., Krienglstein J. (1991). Neuroblastoma cells for testing neuroprotective drug effects. J. Pharmacol. Methods.

[36] Phaisan S., Yusakul G., Sakdamas A., Taluengjit N., Sakamoto S., Putalun W. (2020). A green and effective method using oils to remove chlorophyll from *Chromolaena odorata* (L.) RM King & H. Rob. Songklanakarin J. Sci. Technol..

[37] Wenk G.L. (1988). Amnesia and Alzheimer’s disease: Which neurotransmitter system is responsible?. Neurobiol. Aging.

[38] Van Beek A.H., Claassen J.A. (2011). The cerebrovascular role of the cholinergic neural system in Alzheimer’s disease. Behav. Brain Res..

[39] Sharma K. (2019). Cholinesterase inhibitors as Alzheimer’s therapeutics. Mol. Med. Rep..

[40] Khan H., Amin S., Kamal M.A., Patel S. (2018). Flavonoids as acetylcholinesterase inhibitors: Current therapeutic standing and future prospects. Biomed. Pharmacother..

[41] Budni J., Bellettini-Santos T., Mina F., Garcez M.L., Zugno A.I. (2015). The involvement of BDNF, NGF and GDNF in aging and Alzheimer’s disease. Aging Dis..

[42] Zhou J., Yang W.-S., Suo D.-Q., Li Y., Peng L., Xu L.-X., Zeng K.-Y., Ren T., Wang Y., Zhou Y. (2018). Moringa oleifera seed extract alleviates scopolamine-induced learning and memory impairment in mice. Front. Pharmacol..

[43] Yadang F.S.A., Nguezeye Y., Kom C.W., Betote P.H.D., Mamat A., Tchokouaha L.R.Y., Taiwé G.S., Agbor G.A., Bum E.N. (2020). Scopolamine-Induced Memory Impairment in Mice: Neuroprotective Effects of *Carissa edulis* (Forssk.) Valh (Apocynaceae) Aqueous Extract. Int. J. Alzheimers Dis..

[44] Berkeley J.L., Gomeza J., Wess J., Hamilton S.E., Nathanson N.M., Levey A.I. (2001). M1 muscarinic acetylcholine receptors activate extracellular signal-regulated kinase in CA1 pyramidal neurons in mouse hippocampal slices. Mol. Cell. Neurosci..

[45] Lee J.E., Song H.-S., Park M.N., Kim S.-H., Shim B.-S., Kim B. (2018). Ethanol Extract of Oldenlandia diffusa Herba Attenuates Scopolamine-Induced Cognitive Impairments in Mice via Activation of BDNF, P-CREB and Inhibition of Acetylcholinesterase. Int. J. Mol. Sci..

[46] Kim J., Seo Y.H., Kim J., Goo N., Jeong Y., Bae H.J., Jung S.Y., Lee J., Ryu J.H. (2020). Casticin ameliorates scopolamine-induced cognitive dysfunction in mice. J. Ethnopharmacol..

[47] Peng S., Zhang Y., Zhang J., Wang H., Ren B. (2010). ERK in learning and memory: A review of recent research. Int. J. Mol. Sci..

[48] Hämäläinen M., Nieminen R., Vuorela P., Heinonen M., Moilanen E. (2007). Anti-inflammatory effects of flavonoids: Genistein, kaempferol, quercetin, and daidzein inhibit STAT-1 and NF-κB activations, whereas flavone, isorhamnetin, naringenin, and pelargonidin inhibit only NF-κB activation along with their inhibitory effect on iNOS expression and NO production in activated macrophages. Mediat. Inflamm..

[49] Bae H.J., Kim J., Jeon S.J., Kim J., Goo N., Jeong Y., Cho K., Cai M., Jung S.Y., Kwon K.J. (2020). Green tea extract containing enhanced levels of epimerized catechins attenuates scopolamine-induced memory impairment in mice. J. Ethnopharmacol..

[50] Zanwar A.A., Badole S.L., Shende P.S., Hegde M.V., Bodhankar S.L. (2014). Antioxidant role of catechin in health and disease. Polyphenols in Human Health and Disease.

[51] Chobot V., Huber C., Trettenhahn G., Hadacek F. (2009). (±)-catechin: Chemical weapon, antioxidant, or stress regulator?. J. Chem. Ecol..

[52] Andrade J.P., Assunção M. (2015). Green Tea Effects on Age-Related Neurodegeneration: A Focus on Neuronal Plasticity. Diet and Nutrition in Dementia and Cognitive Decline.

[53] Mandel S., Youdim M.B. (2004). Catechin polyphenols: Neurodegeneration and neuroprotection in neurodegenerative diseases. Free Radic. Biol. Med..

[54] Gunesch S., Hoffmann M., Kiermeier C., Fischer W., Pinto A.F., Maurice T., Maher P., Decker M. (2020). 7-O-Esters of taxifolin with pronounced and overadditive effects in neuroprotection, anti-neuroinflammation, and amelioration of short-term memory impairment in vivo. Redox Biol..

[55] Abd El-Razek M.H. (2007). NMR assignments of four catechin epimers. Asian J. Chem..

[56] Cecile C., Stephanie D., Stephane L., Bernadette C., Christian R. (2000). Characterization of methylation site of monomethyl flavan-3-ols by liquid chromatography/electrospray ionization tan-dem mass spectrometry. Rapid Commun. Mass. Spectrom..

[57] Jung W.K., Tae B.K., Heejung Y., Fischer W., Sang H.S. (2016). Phenolic compounds isolated from Opuntia ficus-indica fruits. Nat. Prod. Sci..

[58] Guilin C., Xun L., Flora S., Mingquan G. (2016). Analysis of flavonoids in Rhamnus davurica and its antiproliferative activities. Molecules.

[59] Jing L., Kun J., Li J.W., Guo Y., Jue W., Yang W., Yi B.J., Qing L., Tie J.W. (2018). HPLC–MS/MS determination of flavonoids in Gleditsiae Spina for its quality assessment. J. Sep. Sci..

[60] Deny S., Hasnah M.S., Farediah A., Rasadah M.A., Norio A., Mariko K. (2007). Antioxidant and cytotoxic flavonoids from the flowers of *Melastoma malabathricum* L. Food Chem..

[61] Shun K., Kazuho H., Kiyotaka H., Hiroaki T., Morifumi H. (2017). Identification of Sternbin and Naringenin as Detoxified Metabolites from the Rice Flavanone Phytoalexin Sakuranetin by Pyricularia oryzae. Chem. Biodivers..

