# Supplementary material for: Euonymus alatus Twig Extract Protects against Scopolamine-Induced Changes in Brain and Brain-Derived Cells via Cholinergic and BDNF Pathways
Source: Nutrients. 2022 Dec 27;15(1):128. doi: 10.3390/nu15010128 (PMC9823662; doi:10.3390/nu15010128)

Supplementary Materials

TABLE OF CONTENTS

S1. General information
S2. Characterization data of isolated compounds
S3. References
S4. Copies of ^{1}H and ^{13}C NMR spectra of isolated compounds
S5. Copies of ESI mass of isolated compounds
S6. HPLC analysis of EA twig extracts
S7. Prep. column chromatography of compounds
S8. Neuroprotective effectiveness of EA twig extact 1 and 2
S9. Effect of EA twig extract and its fraction on B35 cell
S10. Figure of mixture effect of EA twig extract 2 and catechin (a) on BDNF and its downstream protein expression in B35 Cells
S11. Set 2 and 3 of Figures, include Figures S16–S18

S.1 General information

The E. alatus twigs were collected in, South Korea, in November 2019, air dried and stored in -20 °C before using for extraction. Two types of ethanol extracts i.e EA twigs extract 1, and EA twigs extract 2 were prepared. The dried twigs of E. alatus twigs (2 kg) were extracted with 70% ethanol (2 × 20 L) for 4 h (2 times) at 80 °C. The extract was filtered through filter paper. The solvent was evaporated under reduced pressure to yield the concentrated twig extract 1 (174 g). HPLC was analyzed with Waters Alliance HPLC system (Waters, Houston, TX, USA) consisting of a binary pump, an online degasser and a diode array detector (DAD). ESI mass spectra were recorded on Waters micromass Z_Q system in the positive or negative mode. ^{1}H and

^{13}C NMR spectra were recorded on varian VNS (600 MHz and 150 MHz, respectively) spectrometers in deuterated acetone. Preparative column chromatography was performed in bioatage skelete preparative chromatography using a C_{18} column. Merck precoated silica gel $F_{254}S$ plates and RP-18 $F_{254}S$ plates were used for thin layer chromatography (TLC). Spots were detected on TLC under UV light or by heating after spraying with Ferric chloride solution.

S2. Characterization data of isolated compounds

Catechin (**a**):[1] white powder; ^{1}H NMR (CD_3COCD_3, 600 MHz): δ 6.76 (1H, d, J = 1.8 Hz), 6.66 (1H, d, J = 7.8 Hz), 6.63 (1H, dd, J = 7.8, 1.8 Hz), 5.89 (1H, d, J = 2.4 Hz), 5.75 (1H, d, J = 1.8 Hz), 4.44 (1H, d, J = 9.0 Hz), 3.89-3.85 (1H, m), 2.79-2.76 (1H, m), 2.42-2.38 (1H, m). ^{13}C NMR (CD_3COCD_3, 150 MHz): δ 156.9, 156.4, 156.0, 144.9, 144.8, 131.3, 119.2, 114.8, 114.3, 99.7, 95.3, 94.5, 81.8, 67.4, 28.0. ESI MS [H^+]: 291, 273, 207, 139, 165, 161.

Epicatechin (**b**):[1] white powder; ^{1}H NMR (CD_3COCD_3, 600 MHz): δ 6.92 (1H, d, J = 1.8 Hz), 6.71 (1H, dd, J = 8.4. 2.4 Hz), 6.65 (1H, d, J = 7.8 Hz), 5.89 (1H, d, J = 1.8 Hz), 5.78 (1H, d, J = 2.4 Hz), 4.74 (1H, s), 4.08-4.06 (1H, m), 2.74-2.70 (1H, m), 2.62-2.58 (1H, m). ESI MS[H^+]: 291, 273, 207, 139, 165, 161.

Aromadendrin (**c**):[2] pale yellowish amorphous powder; ^{1}H NMR (CD_3COCD_3, 600 MHz): δ 11.57 (1H, s), 7.28 (2H, d, J = 8.4 Hz), 6.76 (2H, d, J = 8.4 Hz), 5.82 (2H, dd, J = 26.4, 1.8 Hz), 4.96 (1H, d, J = 12.0 Hz), 4.52 (1H, d, J = 12.0 Hz). ^{13}C NMR (CD_3COCD_3, 150 MHz): δ 197.4, 167.0, 164.1, 163.3, 158.0, 129.4, 128.2, 115.0, 100.6, 96.2, 95.2, 83.5, 72.2. [MS –H]$^-$: 287, 269, 259, 201, 125.

Taxifolin (**d**):[2] pale yellowish amorphous powder; ^{1}H NMR (CD_3COCD_3, 600 MHz): δ 11.60 (1H, s), 6.93 (1H, d, J = 1.8 Hz), 6.77 (1H, dd, J = 7.8, 1.8 Hz), 6.72 (1H, d, J = 7.8 Hz), 5.85 (1H, d, J = 2.4 Hz), 5.81 (1H, d, J = 1.8 Hz), 4.87 (1H, d, J = 11.4 Hz), 4.46 (1H, dd, J = 11.4, 3.0 Hz). $[MS –H]^-$: 303, 285, 275, 257, 177, 161, 125.

Naringenin (**e**):[3] white amorphous powder; ^{1}H NMR (CD_3COCD_3, 600 MHz): δ 12.05 (1H, s), 7.27 (2H, dd, J = 8.4, 3.6 Hz), 6.76 (2H, dd, J = 8.4, 3.6 Hz), 5.82 (2H, d, J = 4.2 Hz), 5.33-5.30 (1H, m), 3.08-3.02 (1H, m), 2.61-2.57 (1H, m). ^{13}C NMR (CD_3COCD_3, 150 MHz): δ 196.4, 166.6, 164.4, 163.5, 157.8, 131.1, 129.9, 128.2, 115.3, 95.9, 95.0, 79.1, 42.6. ESI MS $[H^+]$: 273, 255, 189, 153, 147.

S3. References

1. a. Abd El-Razek, MH. NMR assignments of four catechin epimers. Asian J. Chem, 2007; *19*, 4867; b. Cecile C, Stephanie D, Stephane L., Bernadette. C, Christian R. Characterization of methylation site of monomethyl flavan-3-ols by liquid chromatography/electrospray ionization tandem mass spectrometry. *Rapid Commun. Mass Spectrom*, 2000; *14*(23), 2312-2319.
2. a. Jung WK, Tae BK , Heejung Y, Sang HS. Phenolic compounds isolated from Opuntia ficus-indica fruits. *Nat. Prod. Sci.*, 2016: *22*(2), 117-121; b. Guilin C, Xun L, Flora S, Mingquan G Analysis of flavonoids in Rhamnus davurica and its antiproliferative activities. *Molecules*, 2016; *21*(10), 1275; c. Jing L, Kun J, Li JW, Guo Y, Jue W, Yang W, Yi BJ, Qing L, Tie JW. HPLC–MS/MS determination of flavonoids in Gleditsiae Spina for its quality assessment. *J Sep Sci*, 2018; *41*(8), 1752-1763.
3. a. Deny S, Hasnah MS, Farediah A, Rasadah MA, Norio A, Mariko K. Antioxidant and cytotoxic flavonoids from the flowers of Melastoma malabathricum L. *Food Chemistry*, 2007; *103*(3), 710-716; b. Shun K, Kazuho H, Kiyotaka H, Hiroaki T, Morifumi H. Identification of Sternbin and Naringenin as Detoxified Metabolites from the Rice Flavanone Phytoalexin Sakuranetin by *Pyricularia oryzae*. *Chem. Biodiversity*, 2017; *14*(2), e1600240.

S4. Copies of ^{1}H and ^{13}C NMR spectra of isolated compounds

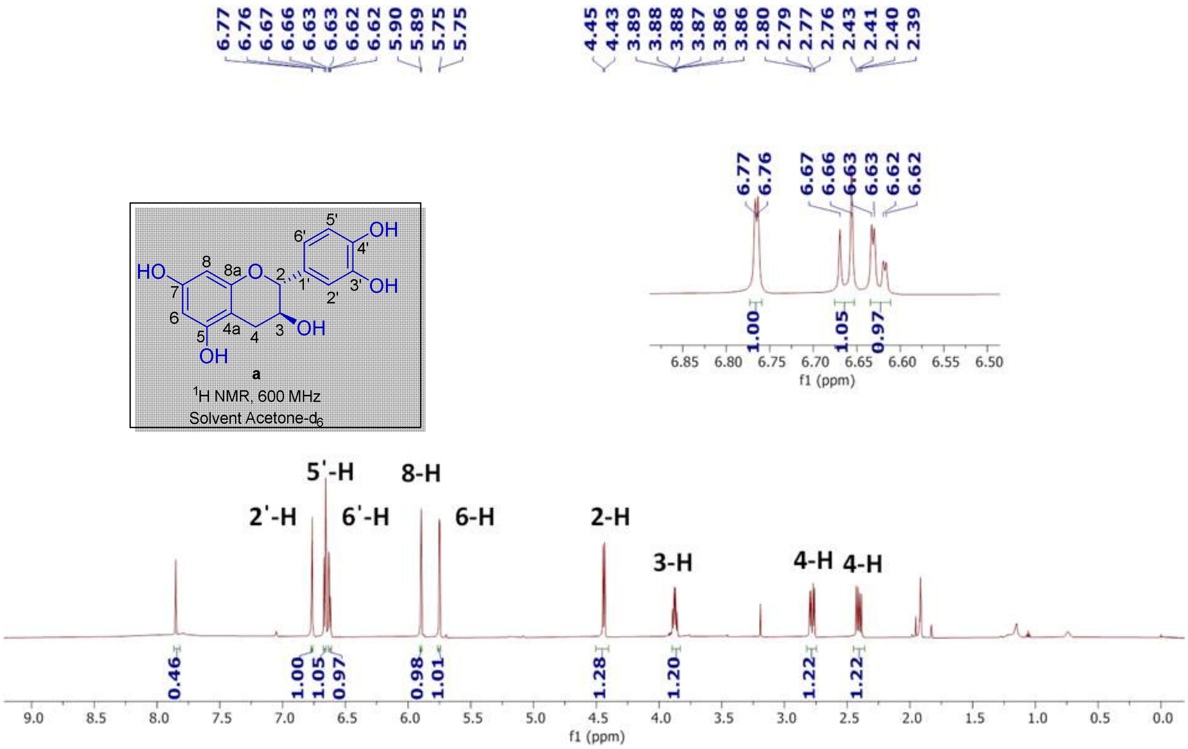

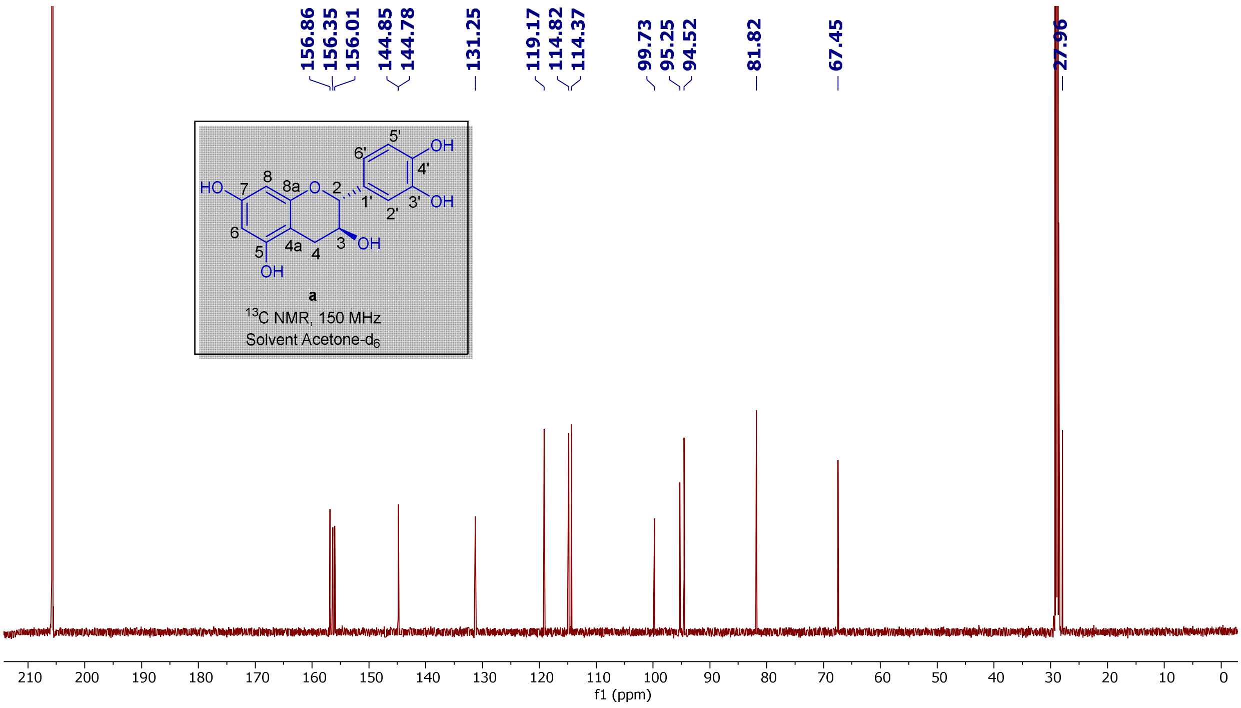

Figure S1. ^{1}H and ^{13}C NMR of catechin (**a**). NMR was analysed in deuterated acetone-d_6.

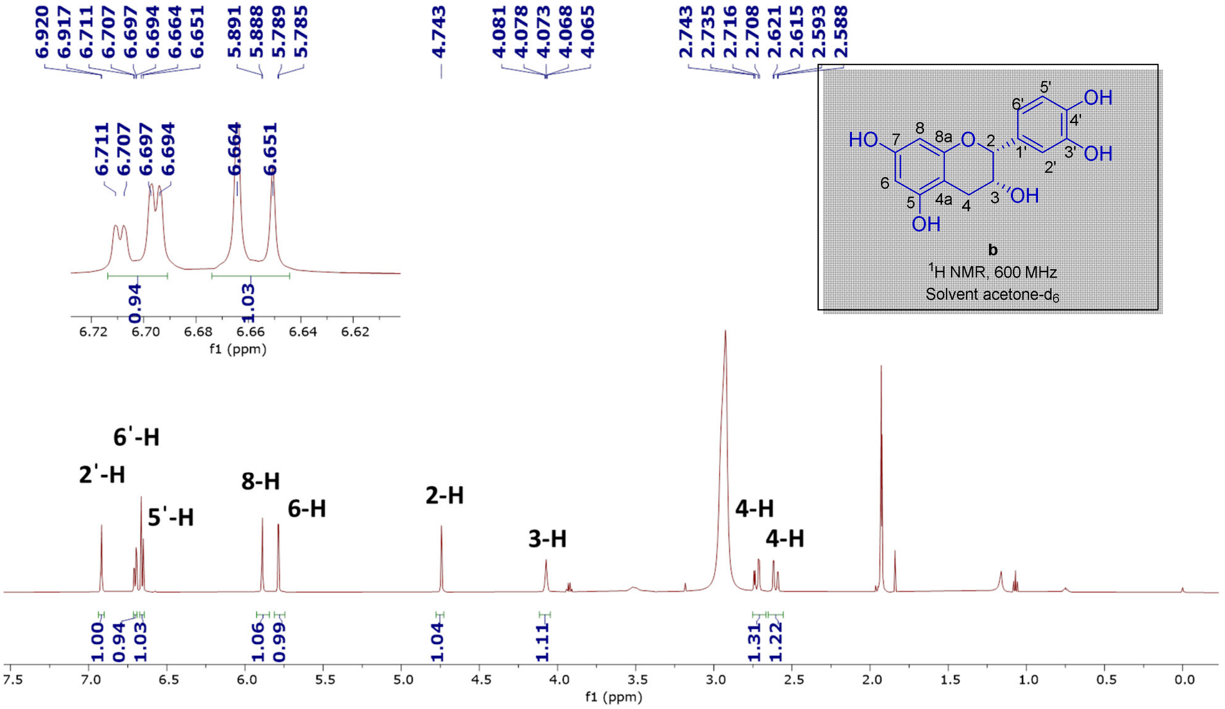

Figure S2. ^{1}H NMR of epicatechin (**b**). NMR was analysed in deuterated acetone-d$_6$.

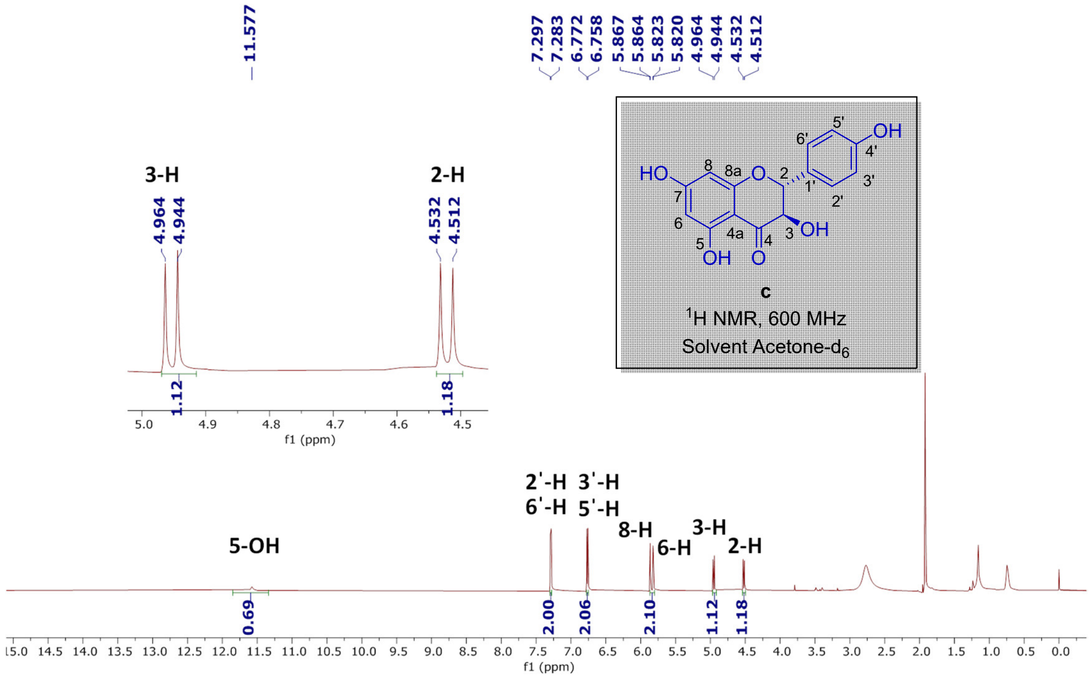

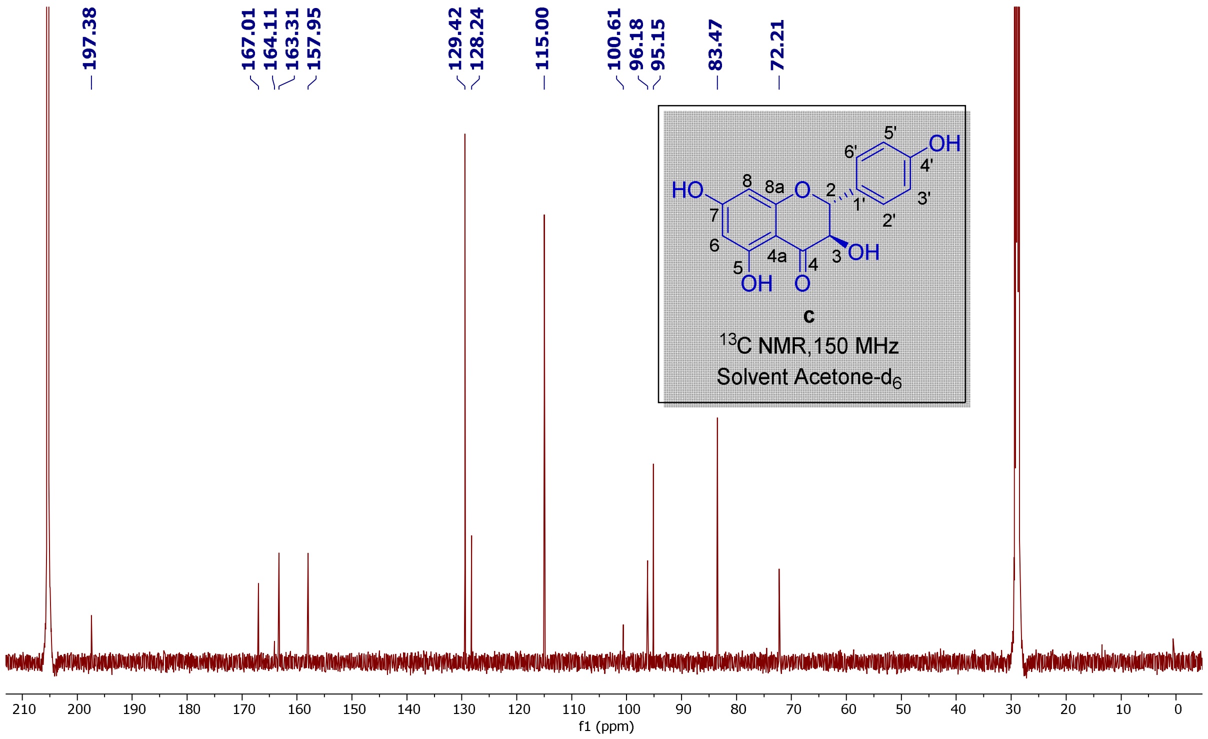

Figure S3. ^{1}H and ^{13}C NMR of aromadendrin (**c**). NMR was analysed in deuterated acetone-d_6.

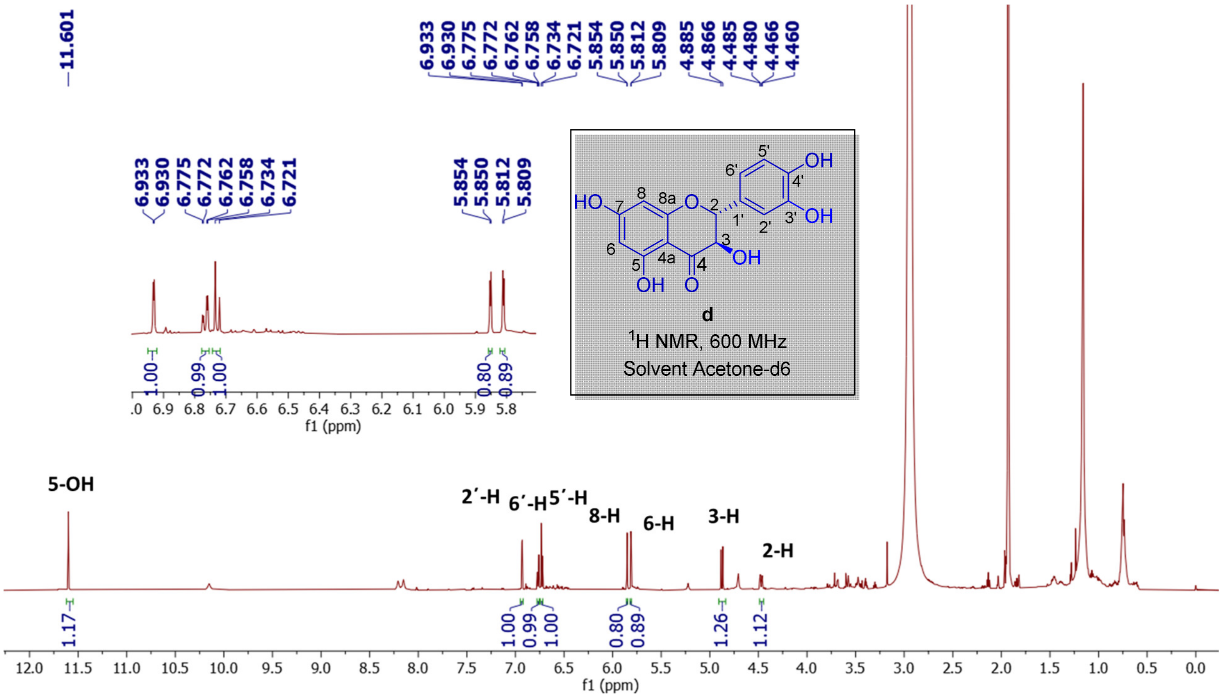

Figure S4. ^{1}H NMR of taxifolin (**d**). NMR was analysed in deuterated acetone-d_6.

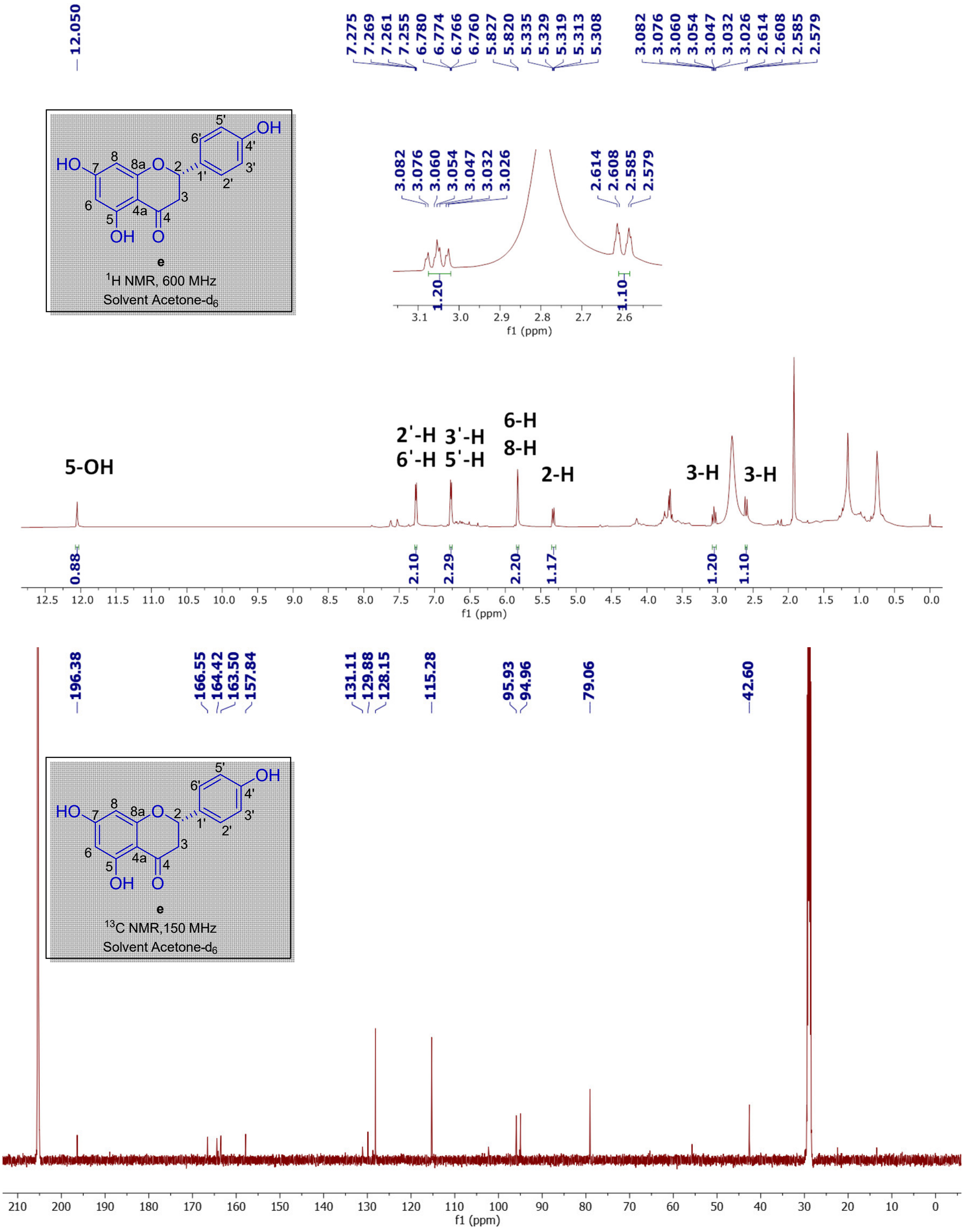

Figure S5. ^{1}H and ^{13}C NMR of naringenin (**e**). NMR was analysed in deuterated acetone-d_6.

S5. Copies of ESI mass of isolated compounds

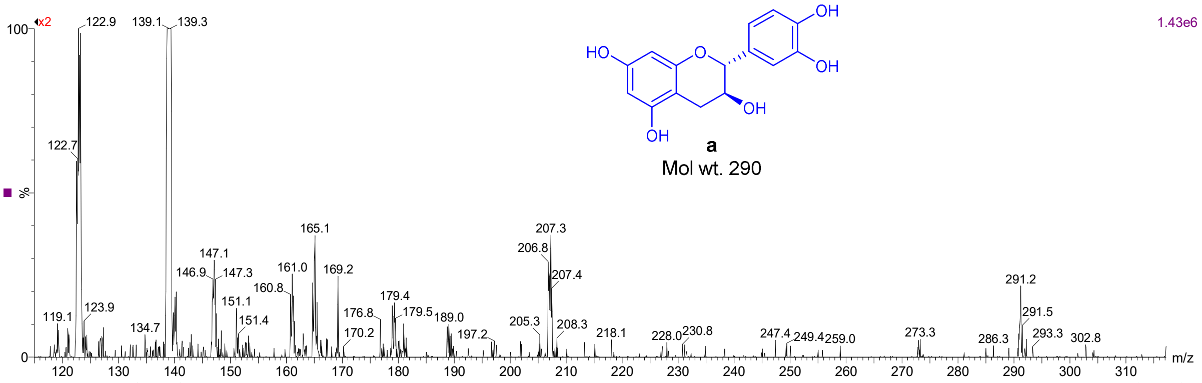

ESI MS H$^+$: 291, 273, 207,179, 165, 151, 147, 139, 123

Figure S6. Positive ESI mass of catechin (**a**).

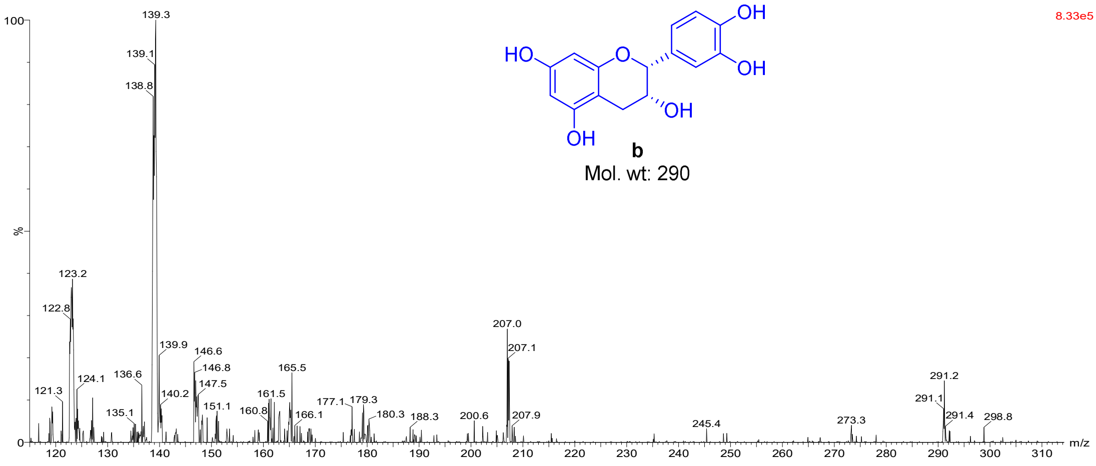

ESI MS H$^+$: 291, 273, 207,179, 165, 151, 147, 139, 12

Figure S7. Positive ESI mass of epicatechin (**b**).

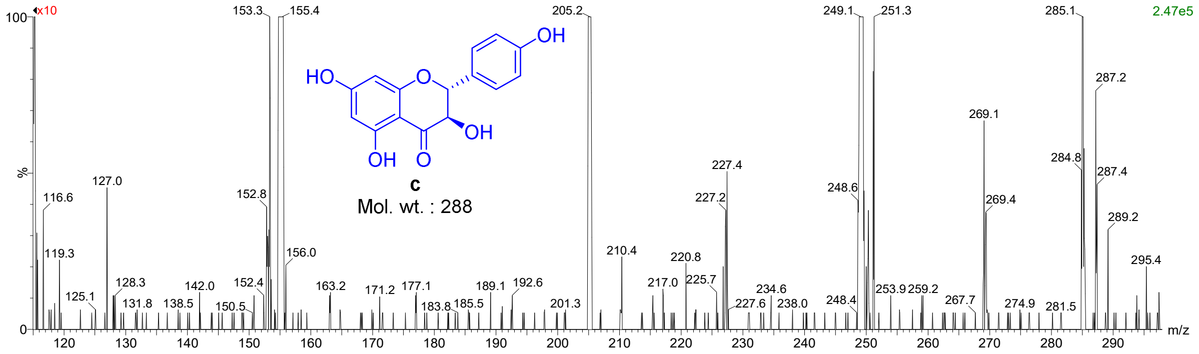

[MS –H]⁻ : 287, 269, 259, 201, 125

Figure S8. Negative ESI mass of aromadendrin (**c**).

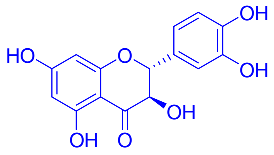

d
Mol. wt. 304

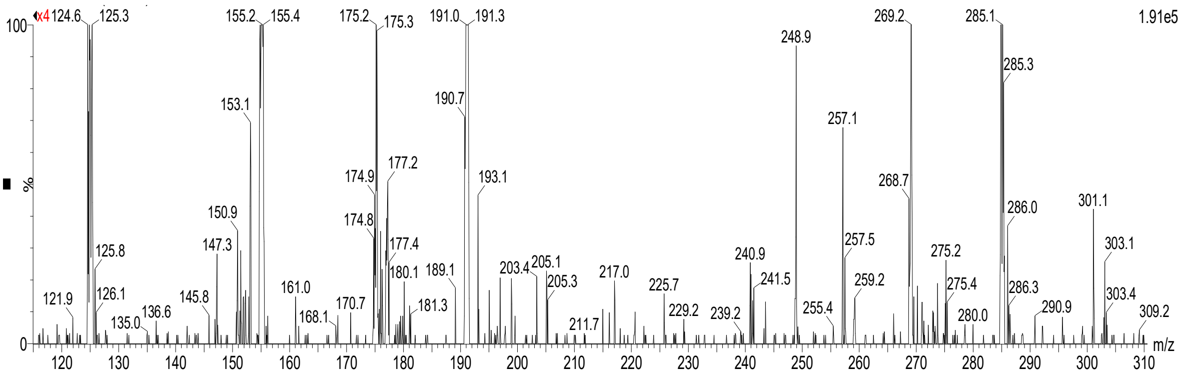

[MS –H]⁻ : 303, 285, 275, 257, 177, 161, 125

Figure S9. Negative ESI mass of taxifolin (**d**).

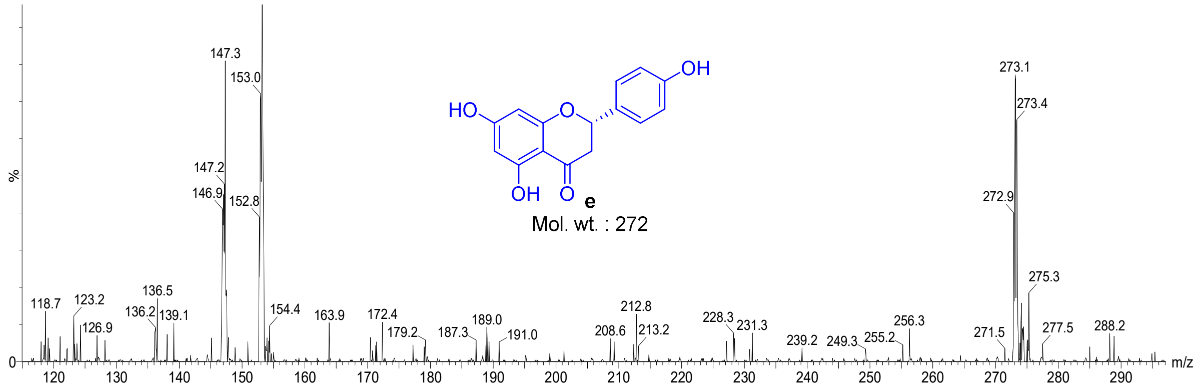

ESI MS H$^+$: 273, 255, 189, 153, 147

Figure S10. Positive ESI mass of naringenin (**e**).

S6. HPLC analysis of EA twig extracts

The HPLC chromatograms of twig extracts 1 and 2 are represented in Figure S11. The UV detection was read at 280 nm. In the chromatogram, five flavonoids were detected: catechin (**a**), epicatechin **(b)**, aromadendrin **(c)**, taxifolin **(d),** and naringenin **(e)**. The peaks were assigned through the isolation of compounds by silica gel chromatography, followed by NMR, ESI MS, and compared with reported journals [S2-S6]. According to the chromatogram and column chromatography, catechin (**a)** was found as a primary compound in twig extracts. In order to aggregate more flavonoids in twig extract, twig extract 2 was made free from primary metabolites such as chlorophyll-containing compounds, oil, lipids, amino acids, etc. However, the major compounds were similar in twig extract 1 and 2.

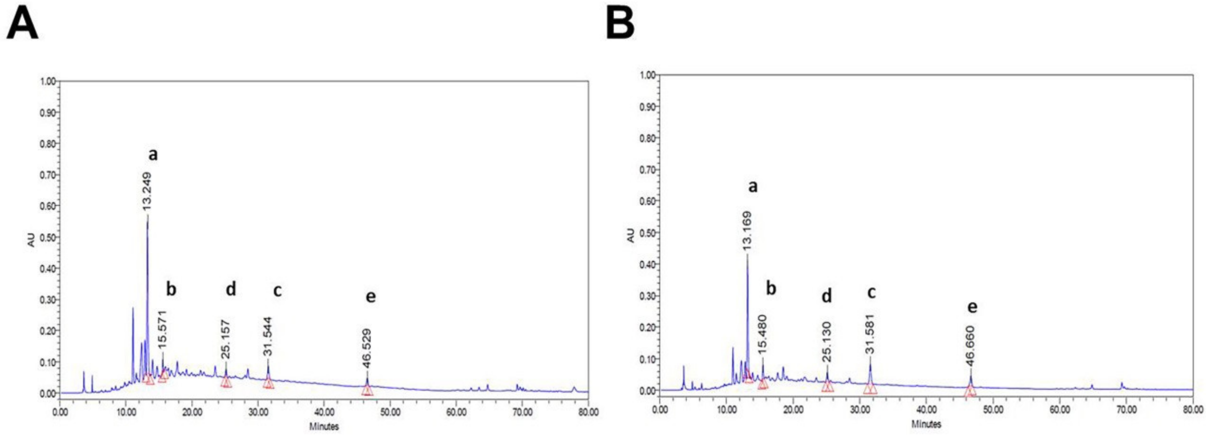

Figure S11. Typical HPLC chromatogram showing the isolated flavonoid's peak of EA twig extract a) 1 and b) 2.

S7. Prep. column chromatography of compounds

Twig extract (2 g) was proceeded to preparative reversed-phase column chromatography using a solvent system of H_2O-MeOH (90:10 to 0:100) to afford three fractions. The fractions were classified into fraction A (Fr.A, 450 mg), B (Fr.B, 300 mg) and C (Fr.C, 150 mg) according to HPLC retention time. Fr.A, B, and C include the major compounds having retention times 0-31 min, 30-50 min, and 50-80 min, respectively. In comparison of three fractions, maximum no. of compounds including compounds catechin (**a**), epicatechin (**b**)**,** aromadendrin (**c**)**,** and taxifolin (**d**) were found in fraction A. In fraction B, fewer compounds were involved, including compounds aromadenrin (**c**) and naringenin (**e**), and fraction C contained mostly non-polar compounds.

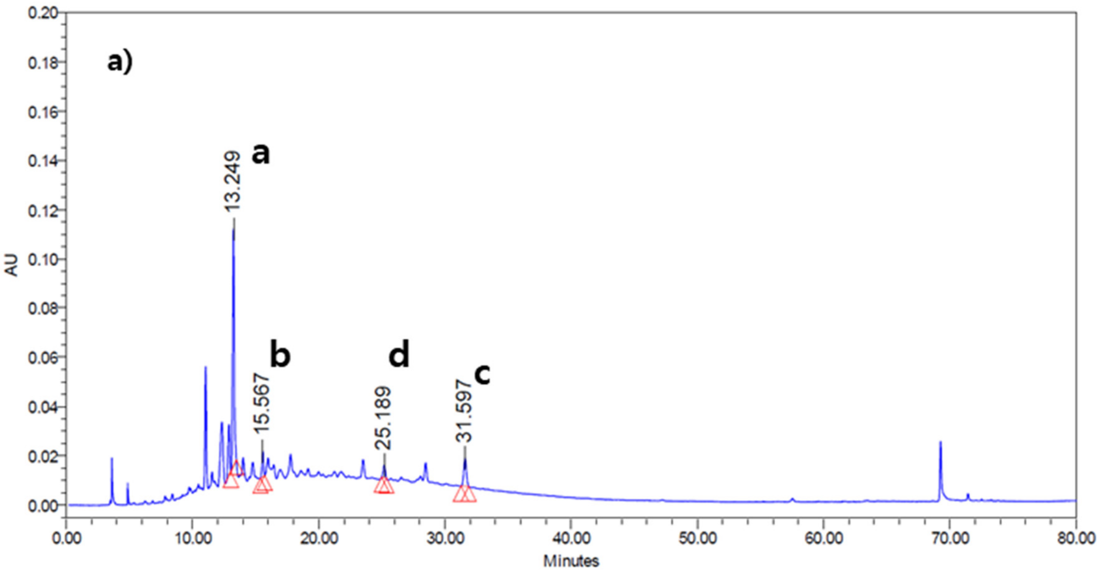

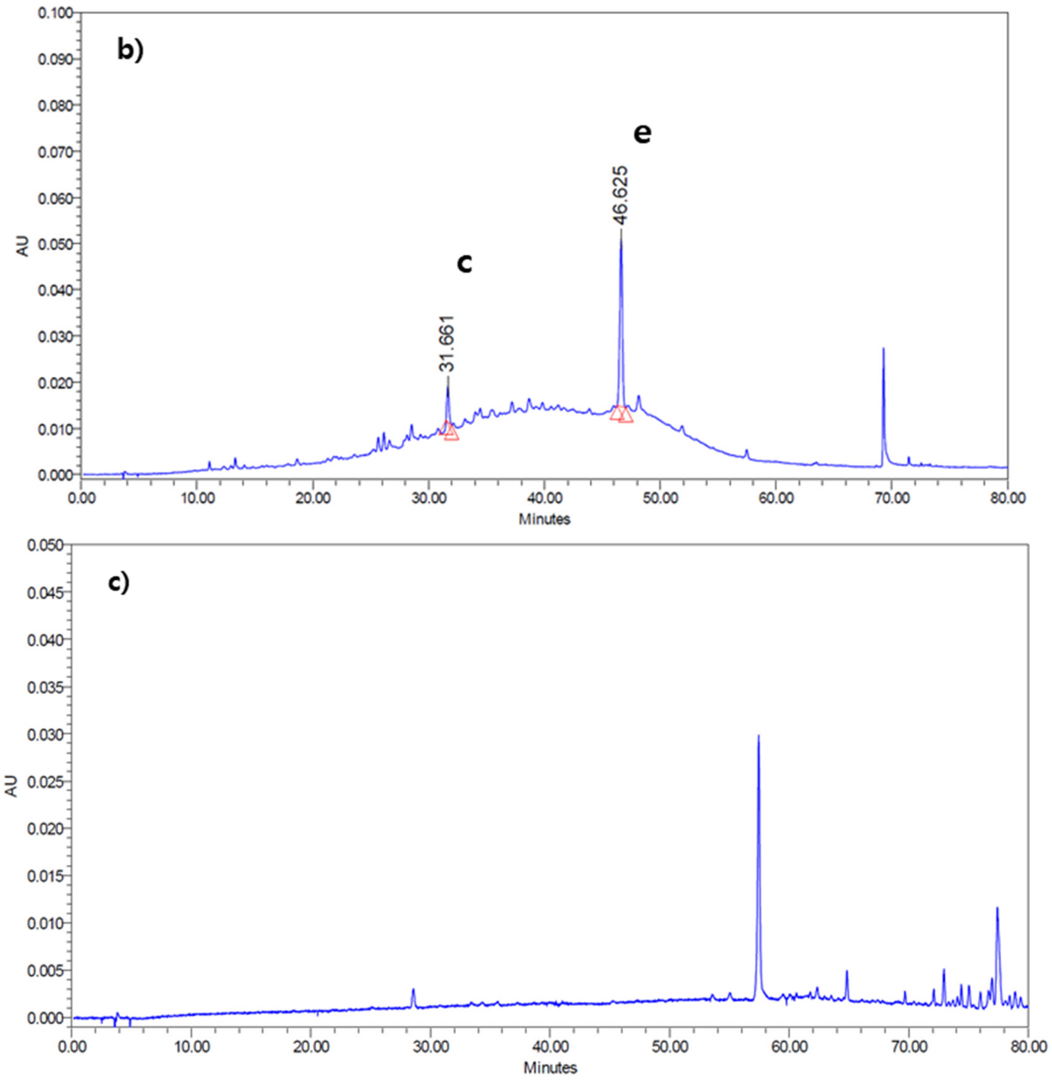

Figure S12. HPLC chromatogram of Fraction A, B and C; a) HPLC chromatogram of Fraction A (280 nm); b) Fraction B (290 nm); c) Fraction C (407 nm).

S8. Neuroprotective effectiveness of EA twig extact 1 and 2

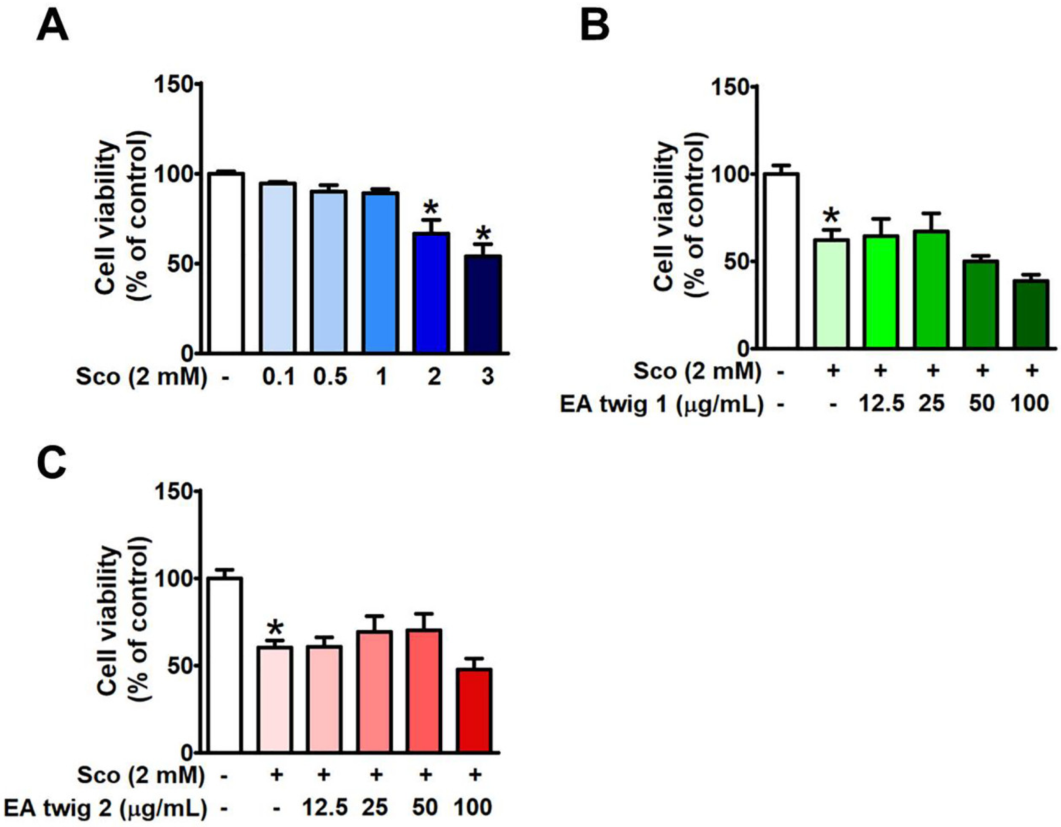

Figure S13. Effects of Scopolamine, EA twig 1, and EA twig 2 extract on B35 cell viabilty. A) Scopolamine decreases cell viability. The cells were exposed to different doses of scopolamine (0.1, 0.5, 1, 2, and 3 mM) for 24 h. The treatment group at 2 and 3 mM showed significant cellular damage. B) The effects of EA twig 1 and C) EA twig 2 extract on scopolamine-mediated cellular damage. The cells were pre-treated with different doses of EA twig 1 and 2 extract (12.5, 25, 50, and 100 μg/mL) for 1 h followed by treatment with scopolamine (2 mM) for 24 h.. Values are expressed as fold of change against the control and were analyzed by one-way ANOVA with Tukey's post-hoc test.*$P < 0.05$, compared with vehicle-treated control group. $^{\#}P < 0.05$, compared with the scopolamine-treated group

.

S9. Effect of EA twig extract and its fraction on B35 cell

Three different fractions (A, B, and C) isolated from EA twig extract through preparative column chromatography were screened for their potential neuroprotective properties against scopolamine-treated B35 cells. Treatment with various fractions of twig extract attenuated the depletion of BDNF, *p*-CREB, and *p*-ERK expressions induced by scopolamine. As shown in Figure S3, Fr. A (100 μg/mL) was exhibited as the most effective fraction with the highest expressions of BDNF, *p*-CREB, and *p*-ERK proteins during scopolamine-induced alterations. Fr. C regained 35% of the decreased level of BDNF, *p*-CREB and *p*-ERK proteins while Fr. B showed the least effect among the three. As a result, the powerful neuroprotective effects of the EA twig extracts might mostly be contributed by constituents in Fr. A.

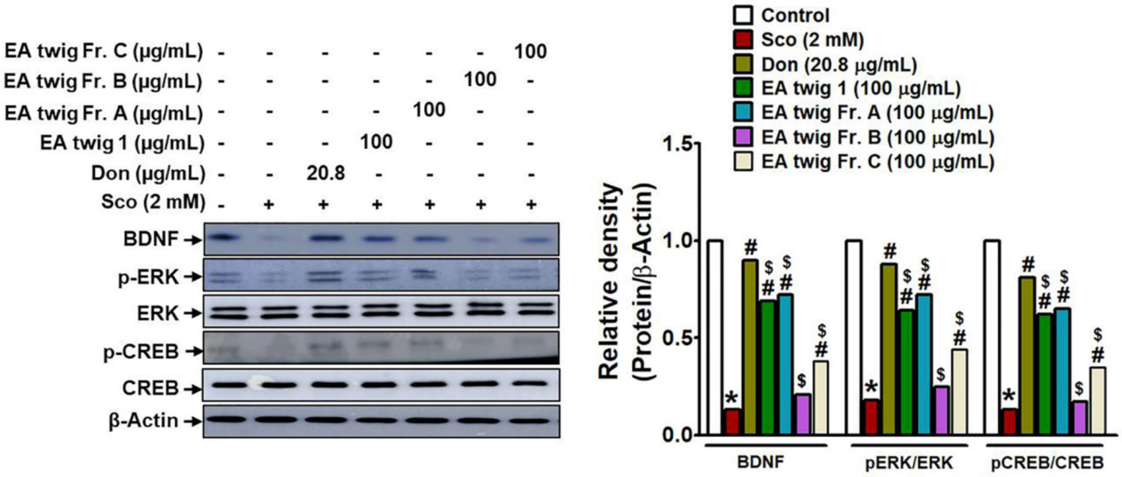

Figure S14. Pretreatment of fractions of EA twig extract reversed scopolamine-induced elevations of BDNF and its down-stream molecules in B35 cells. Cells were pretreated with EA twig extract and their fractions A, B, and C for 1 h followed by treatment with scopolamine for 24 h. Levels of BDNF, *p*-ERK, and *p*-CREB were determined by western blot analysis. Values are expressed as fold of change against the control and were analyzed by Student's t-test. **P* < 0.05, compared with vehicle-treated control group. $^{\#}P < 0.05$, compared with the scopolamine-treated group. $^{\$}P < 0.05$, compared with donepezil-treated group.

S10. Figure of mixture effect of EA twig extract 2 and catechin (a) on BDNF and its downstream protein expression in B35 cells.

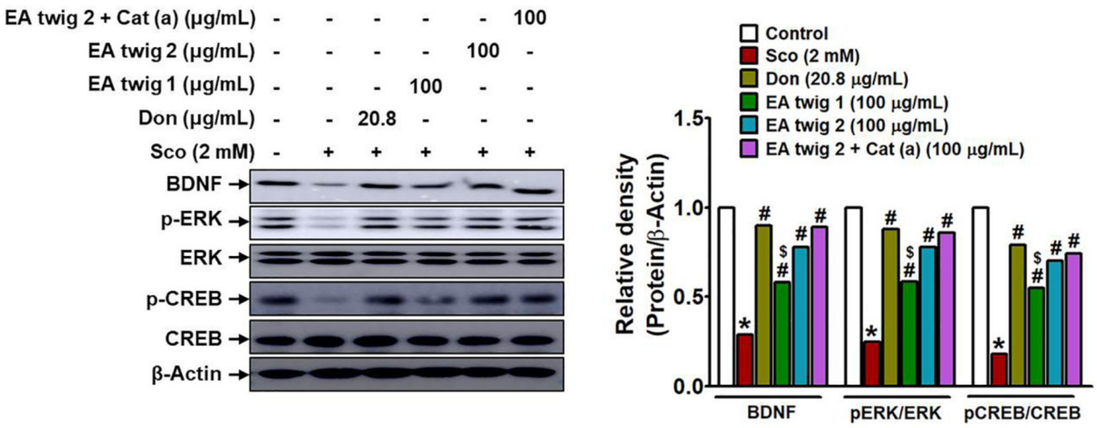

Figure S15. Additive effect of catechin and twig extract combination in BDNF/p-CREB/p-ERK expressions in B35 cells**.** Cells were treated with scopolamine prior to pretreatment with EA twig extract 1, 2 and mixture of **a** and twig extract (100 μg/mL) for 24 h. Western blot analysis was performed to analyze the levels of BDNF, p-ERK and *p*-CREB. Protein expression levels were normalized to those of β-actin and were analyzed by Student's t-test. *$P < 0.05$, compared with vehicle-treated control group. $^{\#}P < 0.05$, compared with the scopolamine-treated group. $^{\$}P < 0.05$, compared with donepezil-treated group

S11. Set 2 and 3 of Figures, include Figures S16–S18.

Figure S16—Set 2 and 3 for Figure 3 in main text

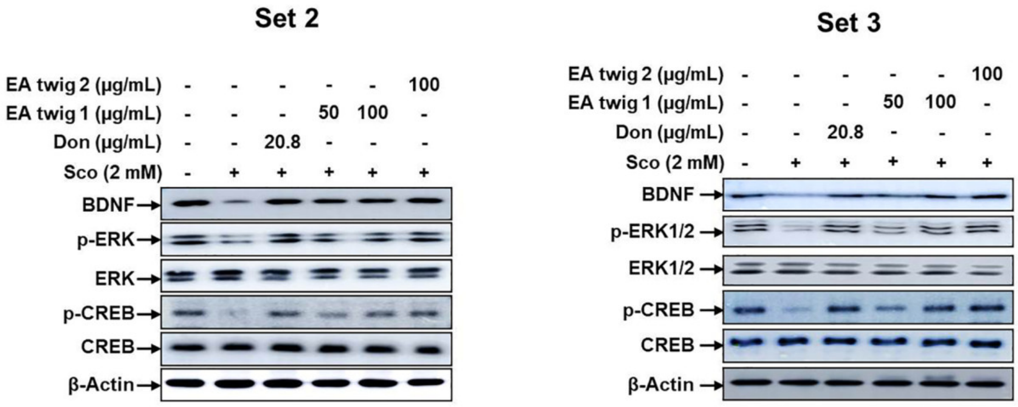

Figure S17A—Set 2 and 3 for Figure 5A in main text

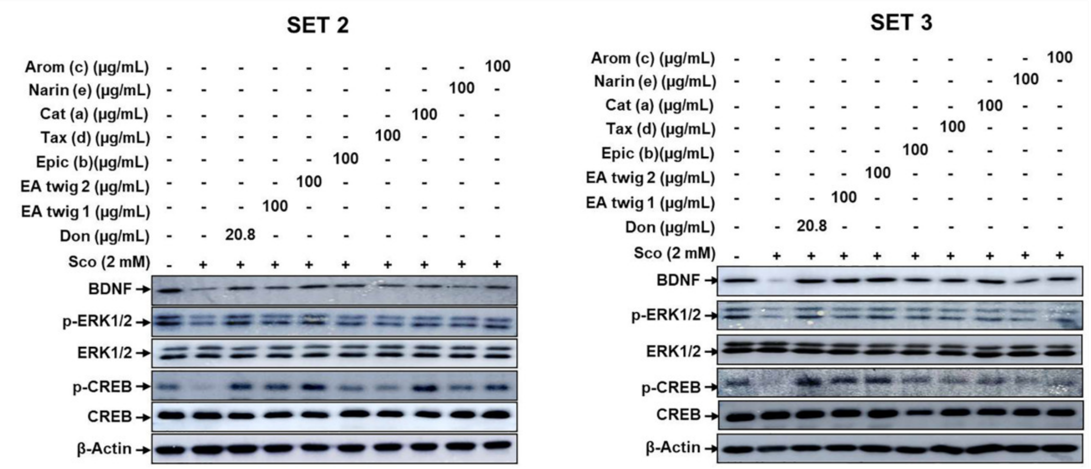

Figure S17B—Set 2 and 3 for Figure 5B in main text

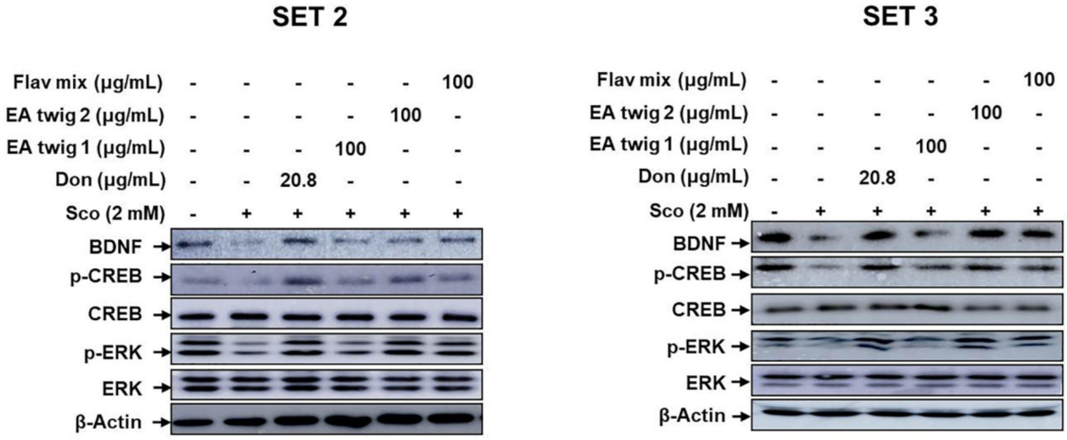

Figure S18—Set 2 and 3 for Figure 7 in main text

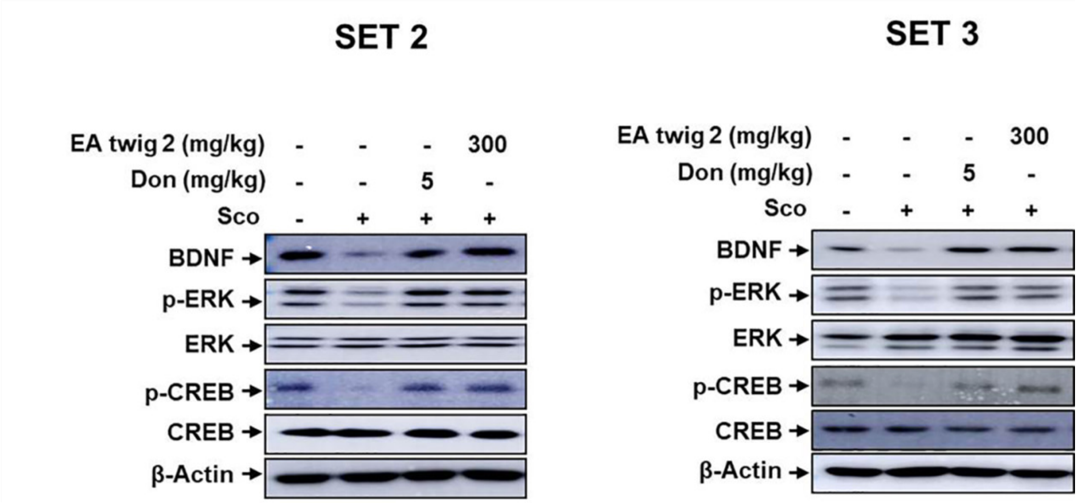

Supplement: Supplementary file 1 [file nutrients-15-00128-s001.zip › nutrients-2044731-supplementary.pdf]
